# Structure-dependent degradation of milk oligosaccharides by newly isolated intestinal commensal bacterial strains from suckling piglets and rabbits

**DOI:** 10.1186/s12866-025-04205-y

**Published:** 2025-08-16

**Authors:** Mathilde Rumeau, Sead Chadi, Frederic Pepke, Martin Beaumont, Cláudia M. Vicente, Agathe Juppeau, Céline Vandecasteele, Laurent Cauquil, Géraldine Pascal, Philippe Langella, Christelle Knudsen, Sylvie Combes, Rebeca Martín

**Affiliations:** 1 GenPhySE, Université de Toulouse, INRAE, ENVT, Castanet- Tolosan, 31326 France; 2 INRAE, AgroParisTech, Micalis Institute, Université Paris-Saclay, Jouy-en-Josas, 78350 France; 3 GeT-PlaGe, INRAE, Genotoul, France Génomique, Université de Toulouse, Castanet Tolosan, 31326 France

**Keywords:** Milk oligosaccharides, Gut microbiota, *Bacteroides*, Glycoside hydrolase, Whole genome sequencing, Short chain fatty acids, Branched chain fatty acids

## Abstract

**Background:**

Mammalian milk oligosaccharides serve as the first natural prebiotics for newborns, promoting the development of a beneficial gut microbiota. The ability of bacteria to use these complex sugars depends on their structure, but data are limited to bacteria isolated from newborn humans. This study aims to investigate *in vitro *the functional relationship between the structural variability of milk oligosaccharides and the metabolic capacities of newly intestinal commensal bacteria isolated from suckling rabbits and piglets.

**Results:**

A total of 240 anaerobic intestinal bacterial strains were isolated from suckling piglets and rabbits, and 9 strains were cultivated in the presence of structurally different milk oligosaccharides: lacto-N-tetraose, 2’-fucosyllactose, and 3’-sialyllactose or 6’-sialyllactose. Five strains, belonging to *Bacteroides fragilis*, *Bacteroides thetaiotaomicron*,* Bacteroides* sp. D2, *Bacteroides* sp. 3_1_33FAA and *Phocaeicola vulgatus* were able to utilize milk oligosaccharides. Growth curves revealed that glucose supported faster growth, while, leading to a lower final biomass compared to milk oligosaccharides. Both the growth rate and the final bacterial biomass varied depending on the milk oligosaccharide structure, with higher final biomass reached with 2’-fucosyllactose. The consumption rates of milk oligosaccharides exceeded 40% for all oligosaccharides in *B. fragilis*, *Bacteroides* sp. 3_1_33FAA and *P. vulgatus* strains. Conversely, *B. thetaiotaomicron* with 6’-sialyllactose and *Bacteroides* sp. D2 strains for each milk oligosaccharide displayed a consumption rate below 40%. Milk oligosaccharide fermentation generated a more diverse metabolome compared to glucose. Utilization of milk oligosaccharides increased the production of propionate, isobutyrate, isovalerate, 2-methylbutyrate and 1,2-propanediol. Remarkably, fermentation of 2’-fucosyllactose resulted in substantial 1,2-propanediol production. Whole genome sequencing of the bacterial strains revealed the presence of diverse glycoside hydrolase in the strains capable of metabolizing milk oligosaccharides.

**Conclusions:**

This study demonstrates the capacity of diverse intestinal commensal bacteria from suckling rabbits and piglets to ferment diverse milk oligosaccharide structures, revealing species-specific and milk oligosaccharide structure-dependent metabolization profiles. These findings highlight the potential application of milk oligosaccharides as prebiotic supplements to support gut health in farm animals.

**Supplementary Information:**

The online version contains supplementary material available at 10.1186/s12866-025-04205-y.

## Background

Milk serves as the primary and sole source of nutrition for mammalian neonates until they can transition to solid food. Lactation is specifically tailored to meet the nutritional and physiological needs of young mammals [1]. Beside nutritional components, milk contains non-nutritional bioactive compounds, such as immunoglobulins, enzymes, antimicrobial peptides, hormones, cytokines and growth factors, which positively influence host physiology and shape the infant’s gut microbiota [2]. Significant attention has been paid to milk oligosaccharides (MOs), the third most abundant component in human milk after lactose and lipids [3]. MOs do not provide nutritional value to the host due to the inability of digestive enzyme to degrade them [4]. Instead, the majority reaches the large intestine intact, where they exert several functions, including the protection against pathogens, the strengthening of the gastrointestinal barrier and the modulation of the immune response [5, 6]. Additionally, MOs are considered as prebiotics, fermentable ingredients selectively used by host gut microbiota resulting in beneficial effects for the host [7]. These molecules are conserved across mammalian species, although variations in their amounts and diversity of structural features have been described [8].

MOs are composed of six monosaccharides residues, glucose (Glc), galactose (Gal), N-acetylglucosamine (GlcNAc), fucose (Fuc), N-acetylneuraminic acid (NeuAc) and N-glycolylneuraminic acid (NeuGc), this last being only present in non-human mammalian milk [9, 10]. The vast majority of MOs begin with the disaccharide lactose at the reducing end (Galβ1-4Glc), which is linked via β1–3 or β1–6 to a disaccharide formed by Gal and GlcNAc. Based on the linkage between these two residues, we distinguish two motifs, lacto-N-biose (Galβ1-3GlcNAc) and N-acetyllactosamine (Galβ1-4GlcNAc) [11]. These motifs can be repeated several times linked via β1–3 or β1–6 [10]. This core can further be elongated by adding one or more Fuc residues linked in α1-2-, α1-3- or α1-4- and/or one or more NeuAc and/or NeuGc linked in α2-3- or α2-6- [11].

Based on the monosaccharide linkage, different glycoside hydrolases (GHs) are required for MO degradation. Consequently, the ability of a bacteria to consume MOs depend on its GH profiles according to the MO structure [12]. The bacterial fermentation of the MOs results in the production of metabolites such as short chain fatty acids (SCFAs), branched chain fatty acids (BCFAs), amino-acid related and nucleic acid-related metabolites [13–15]. These metabolites can shape the gut microbial composition, provide energy to the host cells, enhance the intestinal mucosal barrier and modulate the immune response [16–20]. However, it is still unclear which specific MOs support the growth of individual bacteria. Most studies use bacteria grown on a mixture of MOs, making it difficult to assess the effects of a specific MOs, although variations in bacterial consumption profiles based on MO structure have been observed by studying single MOs [21, 22]. For instance, in humans, *Bifidobacterium* species, which play a crucial role in the gut of human neonates, have received considerable attention due to their strong ability to utilize human MOs (HMOs) [23–28]. However, these bacteria are less prevalent in the gut microbiome of other young mammalian species such as pigs and rabbits [29, 30], and several studies indicated that the utilization of MOs is not exclusive to *Bifidobacteria* members [31, 32].

This study aims to explore *in vitro* the functional relationships between MOs structural diversity and the metabolic capacities of newly isolated commensal bacteria using feces samples of newborn piglets and cecal content samples of newborn rabbits, two species that often encounter digestive disorders around weaning [33, 34]. Effectively managing microbial colonization in these animals in early life is crucial for improving their health [35].

## Materials and methods

### Sample collection

Fresh fecal samples were collected from three 28 days-old healthy Piétrain x (Large White x Landrace) suckling piglets directly from the rectum into anaerobic devices (GutAlive, MicroViable therapeutics; Spain) at INRAE experimental facilities (UE1421 UEPR, Saint-Gilles, France; Agreement No. D35-275-32). The breeding rooms were environmentally controlled to keep the ambient temperature around 24°C. The piglets stayed with their biological mother until weaning (28 days of age). Three 16–23 days-old healthy suckling rabbits were killed by electronarcosis followed by an exsanguination at the INRAE PECTOUL experimental facility (GenPhySE, Castanet-Tolosan, France) (10.17180/ftvh-x393). Caeca were carefully dissected, and their content transferred to anaerobic devices (GutAlive, MicroViable therapeutics). The animals were raised in our INRAE experimental facilities; for pigs: UE1421 UEPR, Saint-Gilles, France; Agreement No. D35-275-32, 10.15454/1.5573932732039927E12, for rabbit: the INRAE PECTOUL experimental facility (GenPhySE, Castanet-Tolosan, France) 10.17180/ftvh-x393). Suckling rabbits were raised with their doe in wire cages with a nest box under control ambient temperature around 24°C. All animal handling was conducted in accordance with the current ethical standards of the European Community (Directive 2010/63/EU), and the French legislation on animal experimentation and ethics. The experiment received the approval of the local ethics committee: Ethical committee in animal experimentation (CEEA) “Science et santé animales CEEA-115” and “Comité Rennais d’Ethique en matière d’Expérimentation Animal CEEA-007” for rabbits and pigs respectively. The approval is registered under the numbers SSA_2021_001 and APAFIS #35552-2022022312418292 v3 for rabbits and pigs, respectively). Details about the sampled individuals, including genetics, sex, age, and body weight are provided in Table S1. All samples were shipped at room temperature and received within 48 h after collection at Commensals and Probiotics-Host Interactions Laboratory (MICALIS Institute INRAE, Jouy- en-Josas, France) where they were stored at 4 °C until processed 24 h later. Samples were introduced in an anaerobic chamber (N_2_ = 90%, CO_2_ = 5% and H_2_ = 5%), weighted, resuspended in physiological serum with 16% glycerol (final concentration) and stored at −80°C.

### Culture media and bacterial isolation

**Fig. 1:**
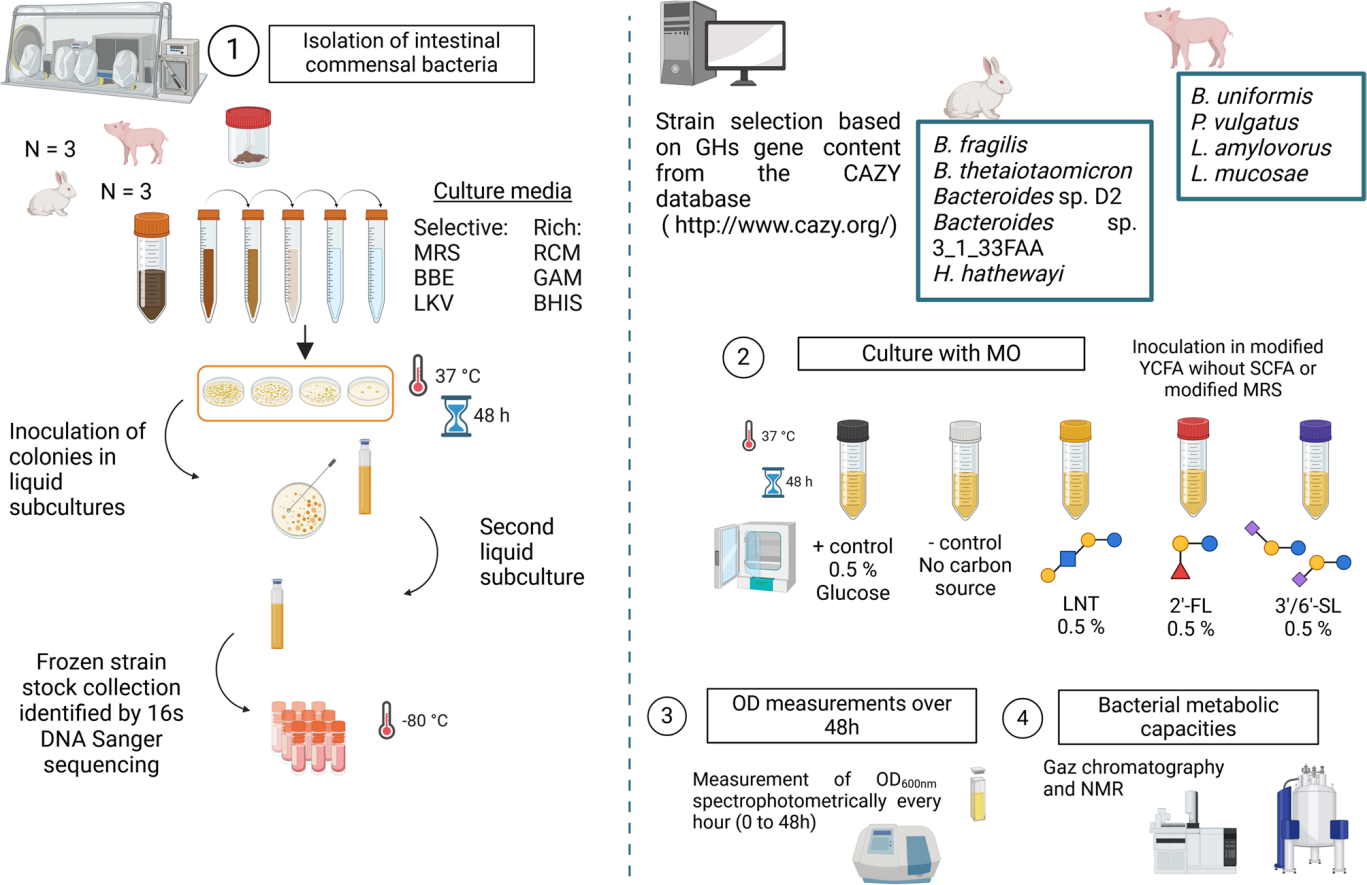
Experimental design for (1) the isolation of intestinal commensal bacteria and the selection of isolates for, (2) the culture of isolated bacteria with MOs, (3) the growth and (4) metabolome analysis after bacterial growth with MOs

Bacterial isolation was performed in an anaerobic chamber with controlled atmosphere (N_2_ = 90%, CO_2_ = 5% and H_2_ = 5%) (Fig. 1). To capture the diversity, three rich culture media were used: Brain heart infusion supplemented with 5 mg/L of hemin (BHIS) (Sigma-Aldrich, Saint-Louis, Missouri, United States); Gifu Anaerobic Medium (GAM) (Himedia, Modautal, Germany); Reinforced Clostridial medium (RCM) (Merck, Darmstadt, Germany) and three selective culture media: Bacteroides bile esculin agar (BBE) (Sigma-Aldrich), selective for the isolation of *Bacteroides*; Laked Brucella Blood Agar supplemented with 10 mg/mL of K1 vitamin, 7.5 mg/L of vancomycin, 100 mg/L of kanamycin and 5 mg/L of hemin (LKV) (Sigma-Aldrich), selective for gram negative *Bacilli*; Man Rogosa and Sharpe (MRS) (Sigma-Aldrich), selective for the isolation of *Lactobacillaceae.* When required, agar (15%, Difco; Franklin Lakes, New Jersey, United States) was added to the media. To reduce the presence of oxygen all the media were supplemented with 0.5 g/L of L-cysteine and introduced in the anaerobic chamber at least 48 h before the experiments.

Samples were homogenized and diluted in physiological serum at a concentration of 100 mg of sample/mL. 100 µL of dilutions 10^−3^ to 10^−6^ were plated. After 48 h of incubation at 37 °C, to maximize variability while minimizing multiple isolations of the same strain, approximately 10 colonies per plate were selected, variable in shape, color and size based on visual observations. Each colony was handpicked and transferred to tubes containing 5 mL of corresponding liquid media and incubated at 37°C. After 24 h of incubation, 100 µL were inoculated in 4 mL of corresponding liquid media and incubated for another 24 h at 37°C. Then, 1 mL of 80% glycerol was added onto the tubes to reach a final concentration of 16% and stored at −80°C. The remaining bacteria in liquid media were removed from the anaerobic chamber for bacterial identification (Fig. 1).

### Enrichment procedure

For rabbit samples, to reduce the presence of *Enterobacteriaceae* and capture the bacterial diversity, culture media for the isolation procedure were supplemented with antibiotics and enrichment methods were also performed. The culture media supplemented were: MRS supplemented with 10 µg/mL of colistin (MRSc); GAM supplemented with 10 µg/mL of colistin (GAMc); BHIS supplemented with 10 µg/mL of colistin and 2 µg/mL of gentamycin (BHISc); LKV; MRS supplemented with 10 µg/mL of colistin and 8 µg/mL of vancomycin (MRS1); MRS supplemented with 4 µg/mL of ampicillin, 10 µg/mL of colistin and 8 µg/mL of vancomycin (MRS2); RCM supplemented with 10 µg/mL of colistin and 8 µg/mL of vancomycin (RCMc). For enrichment methods, 50 µL of frozen rabbit cecal content were incubated for 48 h at 37 °C in 5 mL of four different selective broth culture media. Following enrichment, the isolation procedure was performed as described above.

### Identification of bacteria using 16 S ribosomal gene sequencing

The remaining liquid culture were centrifuged 15 min at 5 000 g to obtain a bacterial pellet. PCR amplification of 16 S rDNA from the pellets was performed in a 25 µL final volume containing 0.2 µM primers, 200 µM dNTP and 0.125 µL of dreamTaq enzyme (Dream Taq polymerase; ThermoFisher Scientific, Waltham, Massachusetts, United States). The amplification program included one cycle at 95 °C for 15 min, followed by 38 cycles composed of 30 s at 98 °C, followed by annealing at 52 °C for 30 s, and extension at 72 °C for 1.5 min. A final extension step at 72 °C for 10 min completed the reaction. Primers used for whole 16 S rRNA gene amplification are listed in Table S2.

After amplification, PCR products were checked using 1% agarose gel. If no amplification was observed, Precellys lysis (3 600 rpm during 30 s three times) was performed on PBS resuspended bacterial pellets with ceramic beads (0–0.25 mm). After a final 5 min centrifugation at 8 000 g, 2 µL of supernatant were used for the amplification as described earlier. Amplification products were sequenced by Eurofins (Nantes, France) with the Sanger sequencing method. The 16 S rDNA sequencing results, aligned on the software BioEdit [36], of approximately 1 000 nucleotides were compared using the EzBiocloud 16 S based ID blast tool (version 20210707) for taxonomic assignment of colonies [37].

### Milk oligosaccharides

Four commercially available MOs representative of the three types of MOs, i.e., lacto-N-tetraose (LNT), 2’-fucosyllactose (2’-FL), 3’-sialyllactose (3’-SL) and 6’-sialyllactose (6’-SL) were supplied by Inbiose (Zwijnaarde, Belgium) (Fig. 1) with purity greater than 90%. The acidic type is represented by two molecules based on the first results of milk oligosaccharide composition in pigs and rabbits, 3’-SL prevailing in pig milk while 6’-SL prevails in rabbits’ [38, unpublished data].

### Selection of isolates to grow with MOs

**Fig. 2:**
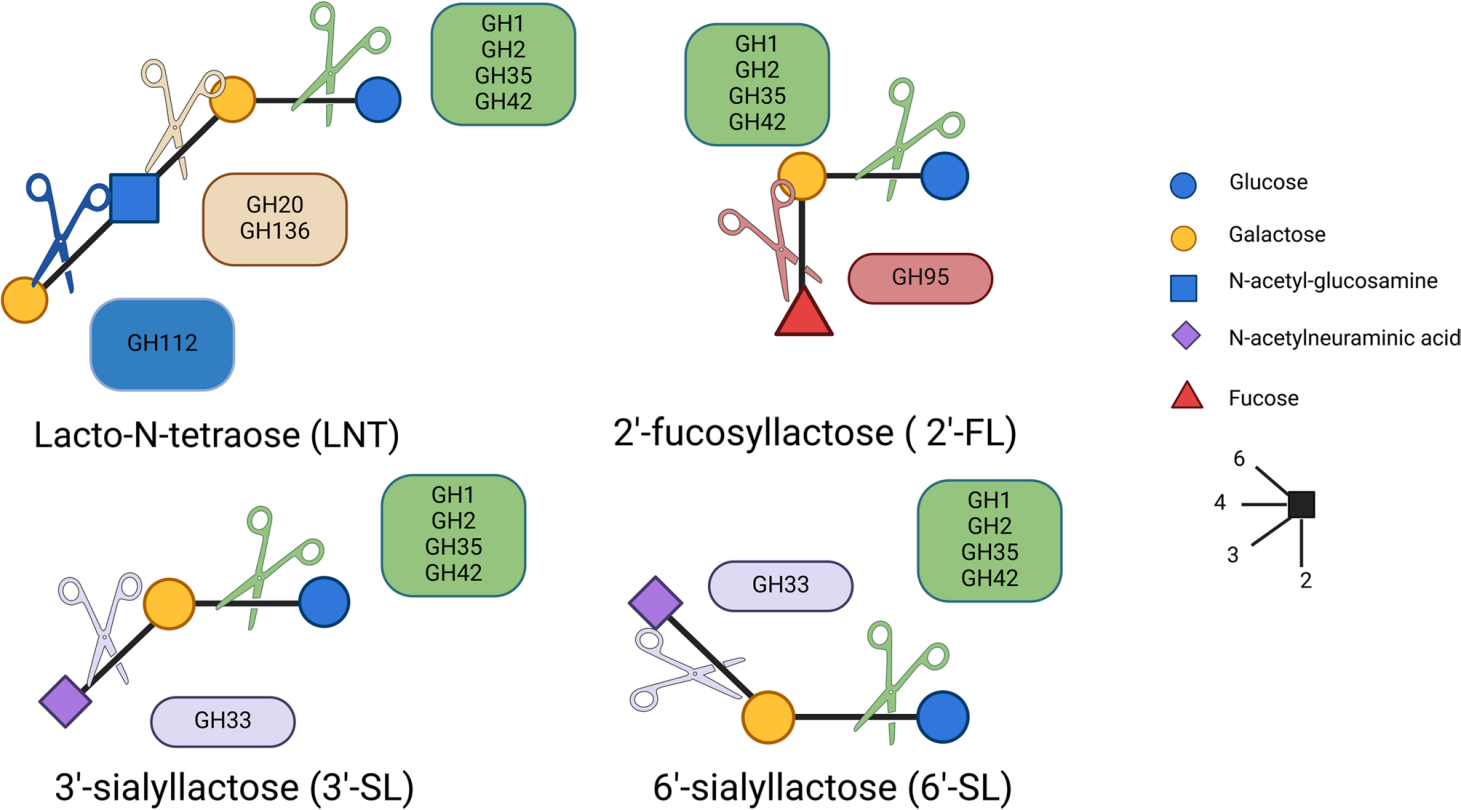
Schematic representation of the structure of neutral (lacto-N-tetraose = LNT), fucosylated (2’-fucosyllactose = 2’-FL) and sialylated (3’-sialyllactose = 3’-SL or 6’- sialyllactose = 6’-SL) MOs along with glycoside hydrolases (GHs) families possibly involved in their hydrolysis. Monosaccharides and linkage representation follow the nomenclature of the Symbol Nomenclature For Glycans (SNFG) [42]. The schema was generated with Biorender

Isolates to grow with the MOs were selected based on the predicted presence of the GH required to hydrolyze the glycosidic linkages of the different MO (Table S3, Fig. 2). The CAZy database [39] was queried using 16S taxonomic affiliations. The enzymes required for the hydrolysis of LNT are classified in the families GH20, GH112 and GH136, the enzymes required for the hydrolysis of 2’-FL are classified in the family GH95 and the enzymes required for the hydrolysis of 3’-SL and 6’-SL are classified in the GH33 family. The enzymes required for the hydrolysis of the remaining lactose, which is shared by the four MOs, are classified in the families GH1, GH2, GH35 and GH42 families [40, 41].

### Capacity of bacteria to degrade MOs

Prior to growth with MOs, bacterial isolates were reconstituted from frozen stocks by preculturing for four days in the appropriate growth media. All experimental steps were carried out in an anaerobic chamber as described above. Frozen stock cultures of the five selected isolates (Isolate_71: *Bacteroides fragilis*, Isolate_128: *Bacteroides thetaiotaomicron*, Isolate_130: *Bacteroides* sp. D2, Isolate_55: *Bacteroides* sp. 3_1_33FAA and Isolate_30: *Hungatella hathewayi*) from suckling rabbit caecum and two isolates (Isolate_182: *Bacteroides uniformis* and Isolate_180: *Phocaeicola vulgatus*) from piglet fecal samples were plated on BHIS agar plates supplemented with 0.5 mg/mL cysteine, 1 mg/mL maltose, 1 mg/mL cellobiose and incubated for 48 h at 37°C. Frozen stock cultures of two isolates (Isolate_183: *Lactobacillus amylovorus* and Isolate_190: *Limosilactobacillus mucosae*) from piglet fecal samples were plated on MRS supplemented with 0.5 mg/mL cysteine and incubated for 48 h at 37°C. Next, colonies were then inoculated in 5 mL of corresponding broth media at 37°C. After 24 h, 100 µL were inoculated in 10 mL of the corresponding broth media and grown for an additional 24 h at 37°C.

After growth, bacteria were centrifuged and the pellets were washed twice in 2 mL of phosphate-buffered saline (PBS). OD_600nm_ of the resulting bacterial suspension in PBS was measured. Two semi-defined media were used for cultivation with MOs, depending on the strain: modified Yeast extract casitone and fatty acid broth (mYCFA) medium [43] lacking SCFAs for *B. fragilis*, *B. thetaiotaomicron*, *Bacteroides* sp. D2, *Bacteroides* sp. 3_1_33FAA, *H. hathewayi*,* B. uniformis* and *P. vulgatus* strains; modified MRS (mMRS) medium [44] for *L. amylovorus* and *L. mucosae* strains. Modified YCFA was prepared, and pH adjusted to 5.90 before autoclaving to obtain a final pH of 6.5. Semi-defined media, mYCFA and mMRS, were supplemented with 0.5% (5 mg/mL) of LNT, 2’-FL, 3’-SL or 6’-SL as the sole carbon source for growth and inoculated with a volume of the bacterial preculture corresponding to 0.1 OD in a final volume of 15 mL as timepoint 0 and incubated anaerobically at 37 °C for 48 h. Media containing 0.5% glucose served as a positive control while media without any carbohydrate source for growth served as a negative control. Bacterial biomass was assessed spectrophotometrically by measuring optical density at 600 nm (OD_600nm_) throughout fermentation (0 to 72 h). The spectrophotometer was blanked with the semi-defined media without bacteria (Fig. 1). Regular plating on Petri dishes with the media used for preculturing (BHIS or MRS) was made to ensure the absence of contamination. Experiments were performed in duplicates or triplicates. During the stationary phase of each bacterial growth, 1.5 mL of liquid culture was collected and centrifuged at 12 000 g at 4 °C for 15 min outside of the anaerobic chamber. Supernatants were recovered and stored at −80°C until analysis (Fig. 1).

### Short and branched chain fatty acids analysis

Concentrations of SCFAs and BCFAs were determined using a gas chromatograph (GC; Agilent 6890 N Network; Santa Clara, California, United States). Frozen supernatants were thawed and deproteinized overnight at 4 °C with the addition of phosphotungstic acid (10% (v/v); Sigma-Aldrich). Samples were then centrifuged for 15 min at 12 000 g at 4 °C and 40 µL of supernatant was added in a 2 mL vial with 10 µL of 2ethyl butyrate (2-ethylbutyric acid, 99%; Sigma-Aldrich) to a final concentration of 20 mM as internal standard.

Two technical replicates were used for each biological replicate. Data were processed using the OpenLab ChemStation software (Agilent) and expressed in mM. Concentrations of SCFA in media before inoculation (timepoint 0) were subtracted from concentrations in samples. Samples with concentrations below quantification limits (0.1 mM for acetate; 0.05 mM for propionate; 0.03 mM for iso butyrate and butyrate; 0.025 mM for isovalerate and valerate) were replaced by the value of their respective quantification limit. Results were expressed as foldchanges relative to the concentration of each metabolite in the culture medium of the same strain cultured with glucose.

### ^1^H-nuclear magnetic resonance spectroscopy analysis

Bacterial supernatants were thawed and centrifuged twice (18 000 g, 4 °C, 10 min). The resulting supernatants (50 µL) were transferred in 600 µL ^1^H-NMR buffer (prepared in D2O, pH 7, TSP 1 mM) and vortexed. Then, 600 µL of the mix were transferred to a 5 mm ^1^H-NMR tube. Samples were analyzed with an Avance III HD NMR spectrometer operating at 600.13 MHz for 1H resonance frequency using a 5 mm inverse detection CryoProbe (Bruker Biospin, Rheinstetten, Germany) in the MetaToul-Axiom metabolomics platform (INRAE, Toulouse, France). After baseline correction and water region (4.5–5.1 ppm) exclusion, spectra (0.5-9 ppm) were bucketed (0.01 ppm bucket width) and normalized by the total area with the R package ASICS [45]. For metabolite identification, spectra of pure compounds prepared in the same buffer and acquired with the same spectrometer were overlaid with sample spectra using Topspin. For each identified metabolite, a bucket nonoverlapping with other metabolites was selected for the quantification as indicated in Table S4. Results were expressed as foldchanges relative to the concentration of each metabolite in the culture medium of the same strain cultured with glucose.

### DNA extraction and whole genome sequencing

Frozen stock cultures of the five selected isolated strains (*B. fragilis*, *B. thetaiotaomicron*, *Bacteroides* sp. D2, *Bacteroides* sp. 3_1_33FAA and *H. hathewayi*) from suckling rabbit caecum and three isolated strains (*P. vulgatus*, *L. amylovorus* and *L*. *mucosae*) from piglet fecal samples were grown anaerobically. A 50 mL aliquot of the enriched culture was prepared and genomic DNA was isolated and purified using the Genomic-tip 100/G kit (Qiagen, Hilden, Germany) according to the manufacturer instructions. DNA extraction was unsuccessful for *B. uniformis* because of technical issues and was not included in the whole genome sequencing. The concentration and purity of the DNA was determined by measuring the absorbance at 230, 260 and 280 nm using a Nanodrop spectrophotometer (Nanodrop-8000; ThermoFisher Scientific). The length of the purified DNA fragments was measured with a Femto^®^ Pulse system (Agilent) electropherogram. Genome sequencing was performed at the GeT-PlaGe genomic platform (Toulouse, France) using the HiFi Single molecule real-time (SMRT^®^) PacBio HiFi sequencing.

### Genome annotation

The raw reads were *de novo* assembled using SMRTLink [46]. Assembly qualities were evaluated with BUSCO [47]. The average nucleotide identity (ANI) and Tetra-nucleotide correlation were calculated using JSpecies and used to affiliate the strain according to their genome [48]. The identification of CAZymes across the genomes was carried out using dbCAN3 [49]. The assembled contigs were annotated into CAZy families and sub-families. Annotations were considered only if they matched with at least two of the three tools. Sequences are available at European Nucleotide Archive (ENA) repository under the reference PRJEB86776.

### Data analysis

All data analyses were performed using R software (v-4.3.2). Growth measurements were analyzed using the Growthcurver package, which also includes parameters interpretations used for the calculation of generation time [50]. A Principal component analysis (PCA) was performed on the ^1^H-NMR metabolites relative concentration using the mixOmics package [51]. Data from all strains were pooled and statistical analysis were performed using the Kruskal-Wallis test, followed by *post-hoc* pairwise comparisons with Dunn’s test, to assess differences between types of MOs, neutral (LNT), fucosylated (2’-FL) and sialylated (3’- SL or 6’-SL). Within isolates (*n* = 2 or 3 biological replicates) analysis is exclusively descriptive.

## Results

### Isolation of gut commensal bacterial strains

**Fig. 3:**
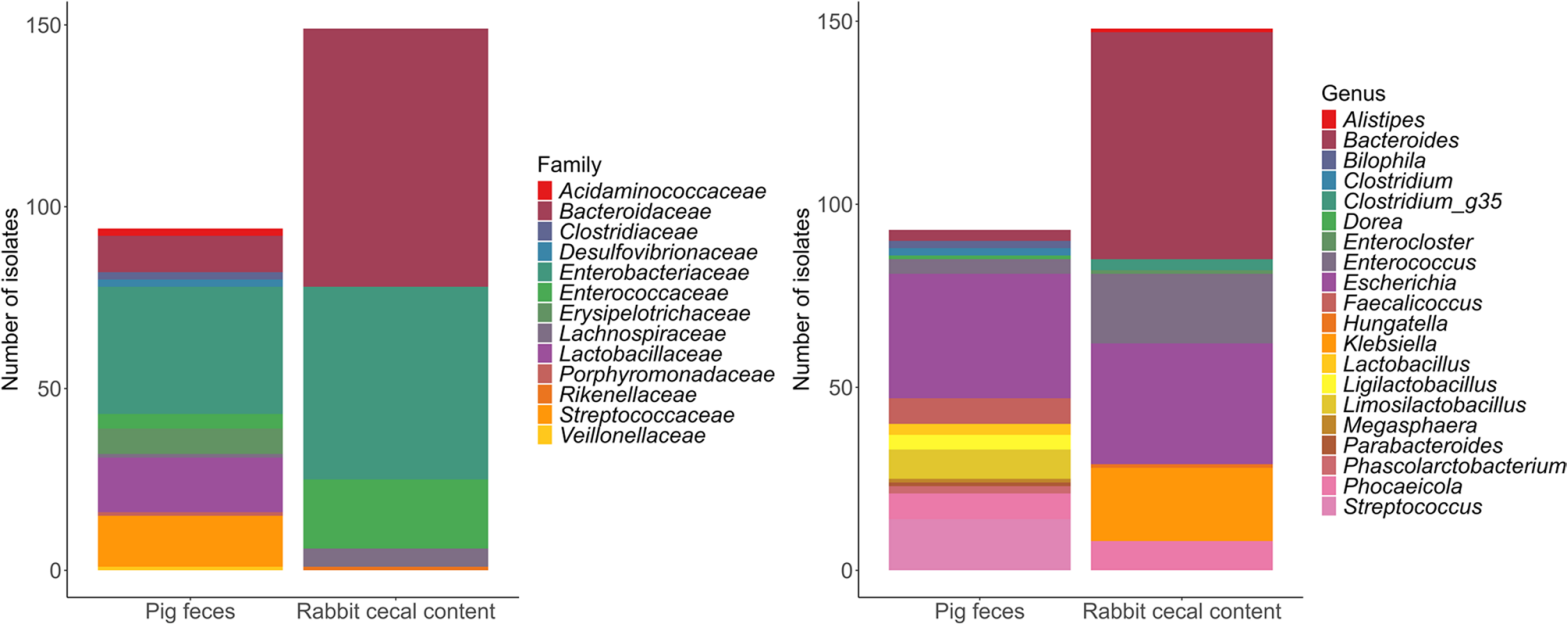
Composition of the collection of bacteria isolated from suckling rabbit caecum and suckling piglet feces at **A** the family level, and at **B** the genus level with data represented as absolute number of isolated strains. Affiliation results are based on the 16 S rDNA sequences using Sanger sequencing using EzBiocloud with a minimum of 92% similarity

To investigate the functional relationships between MOs structural diversity and metabolic capacities of intestinal commensal bacteria, we isolated gut commensal bacterial strains from suckling rabbits and piglets. We targeted bacterial species belonging to dominant families from suckling piglets (*Prevotellaceae*,* Lachnospiraceae*,* Ruminocacaceae* and *Lactobacillaceae*) and suckling rabbits (*Bacteroidaceae*, *Lachnospiraceae* and *Ruminococcaceae*) [30, 52] using three rich and three selective media. After analysis of their 16 S DNA sequence, a collection of 240 unique bacterial isolates was constituted, 148 from suckling rabbit caeca and 92 from suckling piglet feces (Table S3). In both host species, the isolated strains belonged to three phyla, *Bacillota*, formerly *Firmicutes* (49% in pigs and 16% in rabbits), *Pseudomonadota*, formerly *Proteobacteria* (39% in pigs and 36% in rabbits) and *Bacteroidota* (12% in pigs and 48% in rabbits). Most of the strains isolated from piglet feces belonged to *Enterobacteriaceae* (37% of isolated strains), followed by *Lactobacillaceae* (16%) and *Streptococcaceae* (11%). Strains isolated from the rabbit caecum mainly belonged to *Bacteroidaceae* (48%), followed by *Enterobacteriaceae* (36%) and *Enterococcaceae* (13%) (Fig. 3). At genus level, in pigs, *Bacteroides* were less prevalent, covering solely 3% of total isolates, *Escherichia* (37%) and *Streptococcus* (15%) being dominant. The addition of strains from the genera *Lactobacillus*, *Ligilactobacillus* and *Limosilactobacillus*, members of the former genus *Lactobacillus*, represent 16% of the isolates (Fig. 3). In rabbits, 42% of strains isolated from rabbits belonged to *Bacteroides*, notably to *NQMG_s* KFT8, an unclassified *Bacteroides* first isolated in human feces and also known as *Bacteroides sp* KFT8, with 32 isolates out of 148. In total, 26 strains isolated from suckling pig feces represented 14 genera from 12 different families while 17 strains isolated from suckling rabbit caecum were spread across 9 genera from 5 families.

In the unmodified media bacterial isolation, the majority (69%) of the isolates in rabbits belonged to the *Enterobacteriaceae* family and were identified in all media, except LKV (Table S3). Adding colistin in MRS, GAM and BHIS media suppressed the growth of *Enterobacteriaceae*, however, *Enterococcaceae*, that were minimal in the first two isolations (3% of isolates), were numerous in these three colistin supplemented media (21%). The enrichment and the supplementation of the media MRS and RCM with antibiotics– colistin, vancomycin, ampicillin– resulted in 33 isolates of *Bacteroidaceae* and one *Rikenellaceae* (Table S3).

### Strain growth is dependent on MO structure

**Fig. 4:**
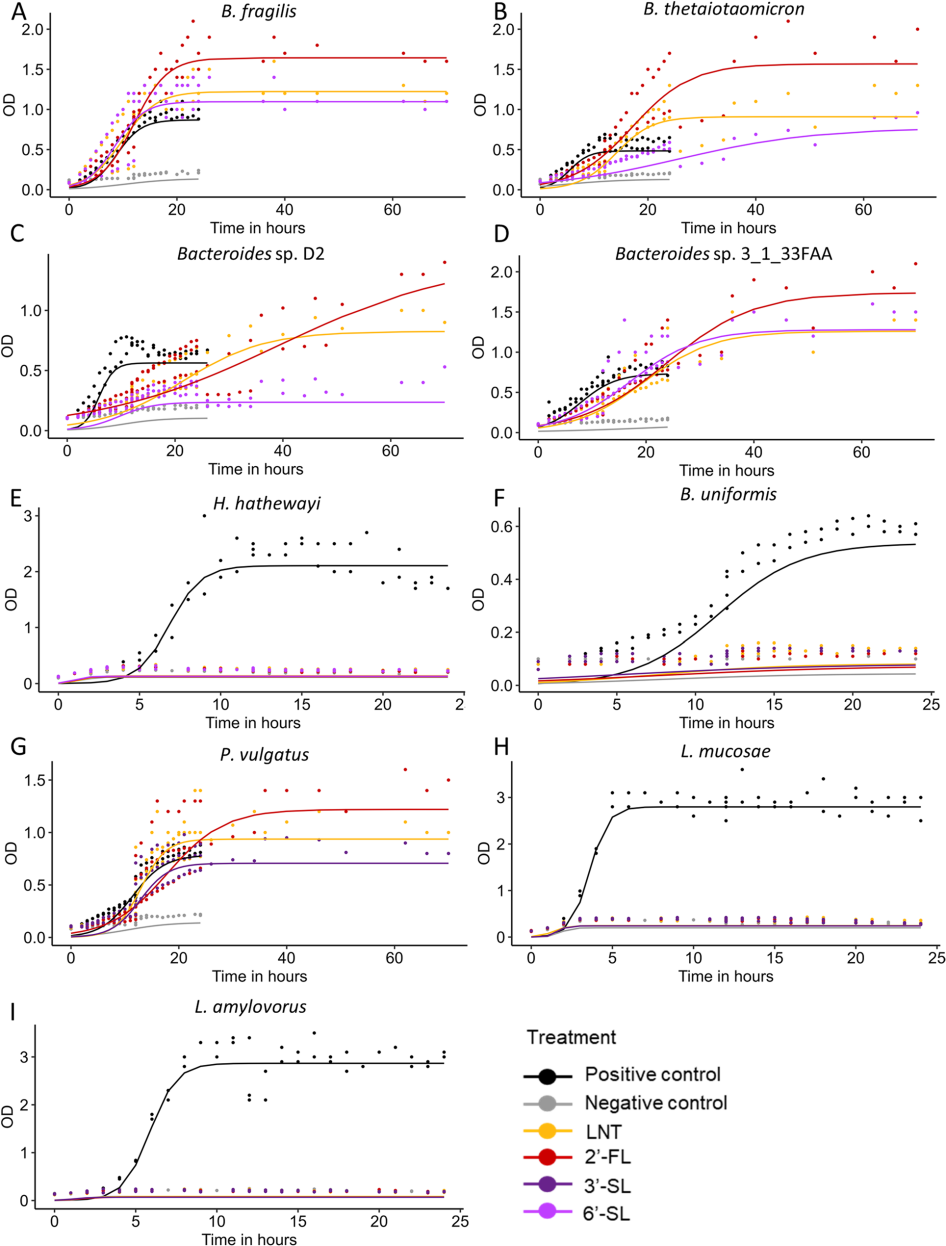
Growth curves of **A** B. fragilis **B** B. thetaiotaomicron **C** Bacteroides sp. D2 **D** Bacteroides sp. 3_1_33FAA **E** H. hathewayi **F** B. uniformis **G** P. vulgatus **H** L. mucosae, and **I** L. amylovorus grown in semi-defined media, mYCFA or mMRS, alone (negative control) or supplemented with either 0.5% glucose (positive control), lacto-N-tetraose (LNT), 2’-fucosyllactose (2’-FL), 3’-sialyllactose (3’-SL) or 6’-sialyllactose (6’-SL). Growth was measured spectrophotometrically over a 72-h period by measuring optical density at a wavelength of 600 nm (OD_600nm_). Curves were generated using the Growthcurver package. Dots represent OD_600nm_ as measured for each replicate (*n* = 2 or 3 per isolate and carbon source), lines represent the predicted values from the logistic regression model. Values are expressed as optical density (OD_600nm_) subtracted by the blank (OD_600nm_ in the medium alone)

Following our investigation of the CAZy database based on 16S taxonomic affiliation, one strain of each species which possess a repertoire of the previously described GHs capable of breaking the bounds found in LNT, 2’-FL, 3’-SL or 6’-SL was selected: *B. fragilis*,* B. thetaiotaomicron*,* Bacteroides* sp. D2 (*NQMG_s* based on 16 S), *Bacteroides* sp. 3_1_33FAA (*P. dorei* based on 16 S), *H. hathewayi*,* B. uniformis*, *P. vulgatus*, *L. amylovorus* and *L. mucosae*. We decided to select *Lactobacillaceae* even though they do not possess many GHs because they are recognized as probiotic strains [53, 54]. To gain more insight into how specific MOs influence the growth of relevant commensal intestinal species, we examined the effects of four MOs representative of the three structural groups, lacto-N-tetraose (LNT), 2’-fucosyllactose (2’-FL), and 3’-sialyllactose or 6’-sialyllactose (3’-SL or 6’-SL for piglet and rabbit isolates respectively). Growth assays were carried out by inoculation in semi-defined media supplemented with 0.5% of each single MO as the only carbon source. Growth pattern with glucose supplemented media was used as reference. Semi-defined media without an added carbon source was used to confirm the inability of our strains to grow solely with the media components. All the selected strains grew with glucose. However, *H. hathewayi*, *B. uniformis*, *L. mucosae* and *L. amylovorus* strains did not grow with any of the MOs, regardless of their structure. In contrast, *B. fragilis*, *B. thetaiotaomicron*, *Bacteroides* sp. D2, *Bacteroides* sp. 3_1_33FAA and *P. vulgatus* strains exhibited a species-specific notable growth with MOs (Fig. 4).

Among the strains that grew with MOs, *B. thetaiotaomicron*, *Bacteroides* sp. D2, *Bacteroides* sp. 3_1_33FAA and *P. vulgatus* strains grew faster when cultivated with glucose compared to MOs, reaching the stationary phase between 15 and 20 h. In contrast, *B. fragilis* reached the stationary phase around 17 h after cultivation for both glucose and MOs. The generation times were shorter with glucose than with MOs, except for *P. vulgatus* when grown with LNT and 3’-SL (Table 1). *B. fragilis* had the shortest generation time with 6’-SL, (0.3 h shorter compared to other MOs). *B. thetaiotaomicron* had the shortest generation time with LNT, 40% shorter than with 2’-FL and 75% shorter than with 6’-SL (Table 1). *Bacteroides* sp. D2 strain showed the shortest generation time with 6’-SL and a 5-time longer generation time with 2’-FL, LNT displaying intermediate values (Table 1). Both *Bacteroides* sp. 3_1_33FAA and *P. vulgatus* strains showed the highest generation time with 2’-FL, 30% longer for *Bacteroides* sp. 3_1_33FAA compared to LNT and 6’-SL and 114% longer for *P. vulgatus* compared to LNT and 3’-SL (Table 1). Although growth was overall fastest with glucose, in most cases the highest OD_600nm_ was reached with MOs (Fig. 4). *B. fragilis* strain achieved 35 (6’-SL) to 84% (2’-FL) higher maximum OD_600nm_ with all three MOs compared to glucose. *B. thetaiotaomicron* strain displayed 61–118% greater maximum growth with 2’-FL compared to LNT and 6’-SL that was only moderately superior to that of glucose (Table 1). Similarly, *Bacteroides* sp. D2 showed delayed degradation with 2’-FL but reached a 40% higher maximal OD_600nm_ compared to LNT. The bacterial biomass of *Bacteroides* sp. D2 with 6’-SL remained low with a maximal OD_600nm_ of 0.53, and did not surpass that of glucose (Table 1). *Bacteroides* sp. 3_1_33FAA demonstrated 88% (6’-SL and LNT) to 147% greater maximum OD_600nm_ with all three MOs compared to glucose. As observed for the other isolates growing with MOs, although slower, the growth with 2’-FL reached a 30% higher maximal biomass compared to LNT and 6’-SL. *P. vulgatus* showed strong growth with LNT and 2’-FL, 26% and 57% higher compared to 3’-SL that induced moderate growth similar to that with glucose (Table 1).

**Table 1:** Metrics associated with the growth curves of the bacteria in semi-defined media, mYCFA or mMRS, supplemented with 0.5% glucose, lacto-N-Tetraose (LNT), 2’-fucosyllactose (2’-FL), 3’-sialyllactose (3’-SL) or 6’-sialyllactose (6’-SL). Growth was measured spectrophotometrically over a 72-h period by measuring optical density at a wavelength of 600 nm (OD_600nm_). Data are presented as mean values (*n* = 2 or 3)

Isolate	Group	Generation time (Hours)	Max OD_600nm_
*Bacteroides fragilis* (Isolate_71)	Glucose	1.58	1.03
	LNT	2.22	1.60
	2’-FL	2.15	1.90
	6’-SL	1.86	1.40
*Bacteroides thetaiotaomicron* (Isolate_128)	Glucose	1.27	0.65
	LNT	2.32	1.30
	2’-FL	3.78	2.10
	6’-SL	8.68	0.96
*Bacteroides* sp. D2 (Isolate_130)	Glucose	0.93	0.75
	LNT	5.26	1.00
	2’-FL	11.7	1.40
	6’-SL	2.24	0.53
*Bacteroides* sp. 3_1_33FAA (Isolate_55)	Glucose	2.33	0.85
	LNT	4.48	1.60
	2’-FL	5.55	2.10
	6’-SL	4.37	1.60
*Hungatella hathewayi* (Isolate_30)	Glucose	0.68	2.70
	LNT	0.35	0.32
	2’-FL	0.26	0.30
	6’-SL	0.27	0.30
*Bacteroides uniformis* (Isolate_180)	Glucose	1.82	0.62
	LNT	2.82	0.14
	2’-FL	4.39	0.13
	3’-SL	4.56	0.14
*Phocaeicola vulgatus* (Isolate_183)	Glucose	2.06	0.85
	LNT	1.62	1.20
	2’-FL	3.65	1.50
	3’-SL	1.82	0.95
*Limosillactobacillus mucosae* (Isolate_190)	Glucose	0.40	3.10
	LNT	0.30	0.39
	2’-FL	0.19	0.40
	3’-SL	0.18	0.40
*Lactobacillus amylovorus* (Isolate_183)	Glucose	0.57	3.40
	LNT	0.50	0.23
	2’-FL	0.45	0.22
	3’-SL	0.35	0.22

In conclusion, although glucose supported faster growth in most cases, the highest biomass production was generally achieved with MOs, particularly 2’-FL. However, there were notable intra-species variations in growth patterns, indicating a structure-dependent preference for certain MOs (Fig. 4).

### Consumption of milk oligosaccharides and accumulation of milk oligosaccharides monosaccharide monomers differ based on the MOs structure

**Fig. 5:**
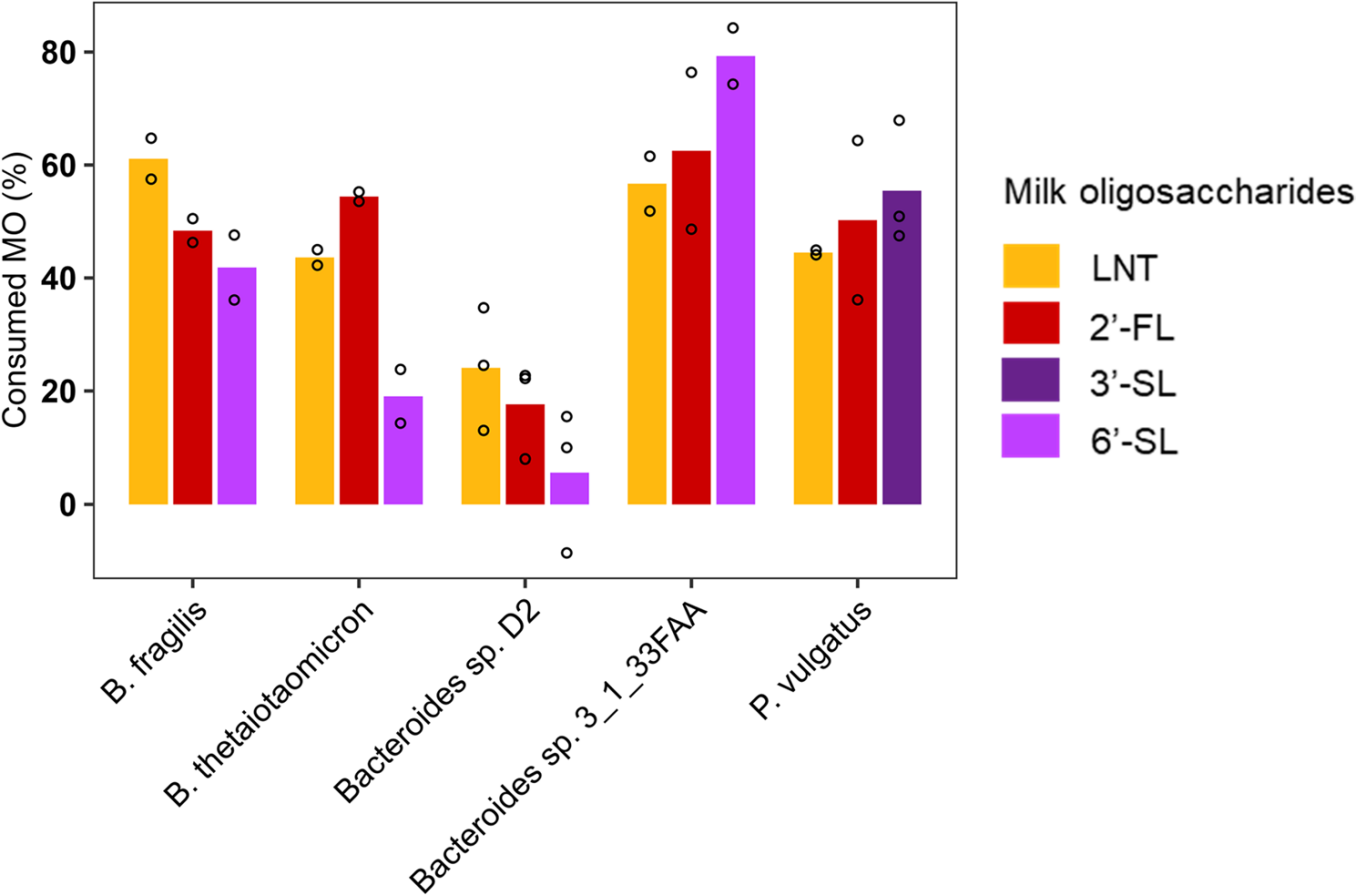
Consumption of substrate after growth in semi-defined media (mYCFA) supplemented with 0.5% lacto-N-tetraose (LNT), 2’-fucosyllactose (2’-FL), 3’-sialyllactose (3’-SL) or 6’-sialyllactose (6’-SL). The concentration was measured by nuclear magnetic resonance during the stationary phase. Data are presented as the relative amount of substrate remaining respectively to the uninoculated medium: YCFA with LNT, 2’-FL, 3’-SL or 6’-SL. Histograms represent mean values and dots represent values of each replicate (*n* = 2 or 3)

**Fig. 6:**
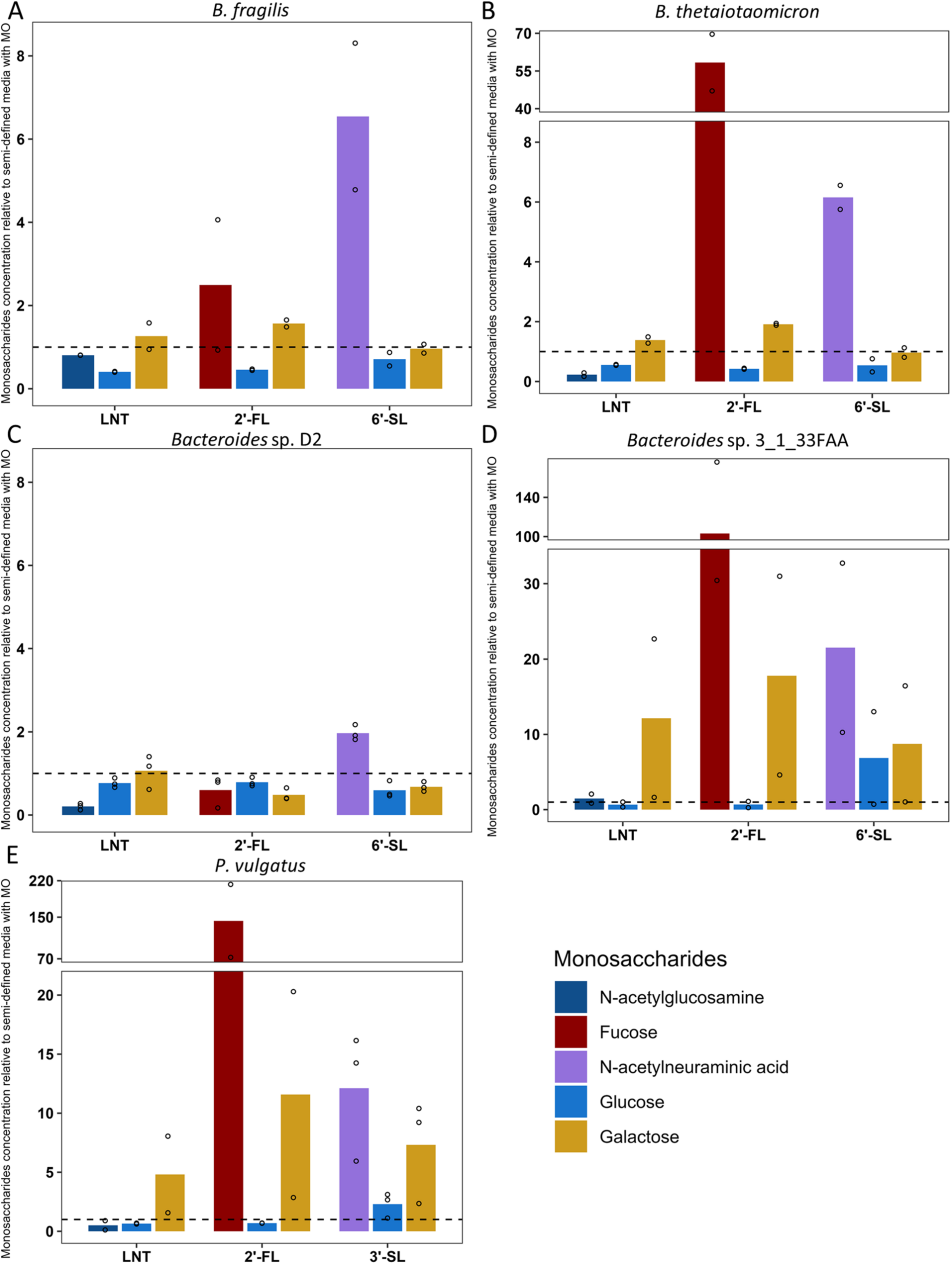
Accumulation of the monosaccharide constituents after growth in semi-defined media (mYCFA) supplemented with 0.5% lacto-N-tetraose (LNT), 2’-fucosyllactose (2’-FL), 3’-sialyllactose (3’-SL) or 6’-sialyllactose (6’-SL) of **A** B. fragilis **B** B. thetaiotaomicron **C** Bacteroides sp. D2 **D** Bacteroides sp. 3_1_33FAA (E) P. vulgatus. Data are presented as the relative amount of monosaccharide remaining respectively to the uninoculated medium: YCFA with LNT, 2’-FL, 3’-SL or 6’-SL. Barplots represent mean values and dots represent values of each replicate (*n* = 2 or 3)

At the end of the culture, the percentage of MOs consumed was calculated (Fig. 5) and the quantities of the MO monomers accumulated in the medium during growth were measured in order to assess whether the bacteria were able to hydrolyze glycosidic links and release monosaccharides (Fig. 6). For all five strains able to degrade MOs, growth resulted in a decrease in the amount of the available MOs, with species-specific consumption patterns observed (Fig. 5). *B. fragilis* strain was efficient at consuming LNT with 61% of the molecules consumed, while only 48% and 41% of 2’-FL and 6’-SL, respectively, were consumed. *B. thetaiotaomicron* strain had the highest consumption for 2’-FL (54%) and consumed 43% of LNT. However, this species was less efficient at consuming 6’-SL with only 19% consumed which is in accordance with growth curves observations. *Bacteroides* sp. D2 was the least efficient in the consumption of all three MOs compared to the other strains, consuming 24% of LNT, 17% of 2’-FL and 5% of 6’-SL. *Bacteroides* sp. 3_1_33FAA was particularly efficient in the consumption of 2’-FL and 6’-SL with 63% and 79%, respectively while consumption of LNT was 57%. *P. vulgatus* consumed all MOs, between 45% and 55% (Fig. 5).

The consumption of MOs can lead to the accumulation of several monosaccharide constituents (Fig. 6). The quantity of GlcNAc, a constituent of the LNT, was not released in the medium with concentrations systematically below those measured in the uninoculated YCFA medium supplemented with LNT. In contrast, the consumption of 2’-FL resulted in the release of a Fuc for *B. thetaiotaomicron*, *Bacteroides* sp. 3_1_33FAA and *P. vulgatus* strains with concentrations 58, 103 and 142-fold-higher than in the uninoculated YCFA medium supplemented with 2’-FL, respectively. For all five strains, NeuAc accumulated in the culture media from the 3’-SL or 6’-SL degradations. This accumulation was however weaker in the supernatants of *Bacteroides* sp. D2 (2-fold-higher), consistent with its low consumption of 6’-SL. *B. fragilis* and *B. thetaiotaomicron* strains released a similar amount of NeuAc, 6-fold-higher than in the uninoculated medium even though percentage of consumption was higher for *B. fragilis* (Fig. 6). *Bacteroides* sp. 3_1_33FAA and *P. vulgatus*, the most efficient at consuming acidic MOs in our experiment, had the highest concentration of NeuAc, from 12-fold-higher for *P. vulgatus* to 21-fold-higher for *Bacteroides* sp. 3_1_33FAA. Irrespective of the milk oligosaccharide’s structure, glucose did not accumulate in the supernatant of *B. fragilis*, *B. thetaiotaomicron* and *Bacteroides* sp. D2 strains with fold change values below 1 (Fig. 6). For galactose, both *B. fragilis* and *B. thetaiotaomicron* displayed concentrations 1 to 2-fold higher when grown with LNT or 2’-FL while the concentration was lower than in the uninoculated medium for 6’-SL. Higher quantities of galactose were detected in the supernatant of *Bacteroides* sp. 3_1_33FAA compared to the media alone with 18-fold higher levels for 2’-FL, 12-fold higher for LNT and 9-fold-higher for 6’-SL. Similarly, concentrations of galactose found within *P. vulgatus* cultures after consumption of 2’-FL, LNT and 3’-SL were 13, 5 and 7-fold higher compared to the uninoculated media respectively (Fig. 6).

### Patterns of SCFA and BCFA production depend on the structure of MOs

We determined the SCFAs and BCFAs (acetate, propionate, butyrate, isobutyrate, isovalerate, valerate, isocaproate and caproate) production by the nine selected strains at the stationary phase during fermentation of LNT, 2’-FL, 3’-SL or 6’-SL, glucose or without supplemented carbon source. Valerate, caproate and isocaproate were undetectable in all the analyzed bacterial supernatants. All the isolates in culture with glucose as carbon source (positive control) produced mainly acetate (Supplementary Fig. 1, Table S5). In line with the inability of *H. hathewayi*, *B. uniformis*, *L. mucosae* and *L. amylovorus* strains to grow with MOs, only minor amounts of acetate were detected when cultured with MOs (< 3.91 mM) while the other SCFAs and BCFAs were not detected in supernatants (Table S5). Regarding the strains able to grow with MOs, *B. fragilis*, *B. thetaiotaomicron*, *Bacteroides* sp. D2, *Bacteroides* sp. 3_1_33FAA and *P. vulgatus* strains displayed a more diverse pattern of SCFAs and BCFAs production when grown with MOs, compared to glucose, with detectable production of acetate, propionate, isobutyrate and isovalerate. Acetate and propionate were the major SCFA products from MOs fermentation while the BCFAs isobutyrate and isovalerate were quantified at lower levels (below 1 mM) (Supplementary Fig. 1 and Table S5).

**Fig. 7:**
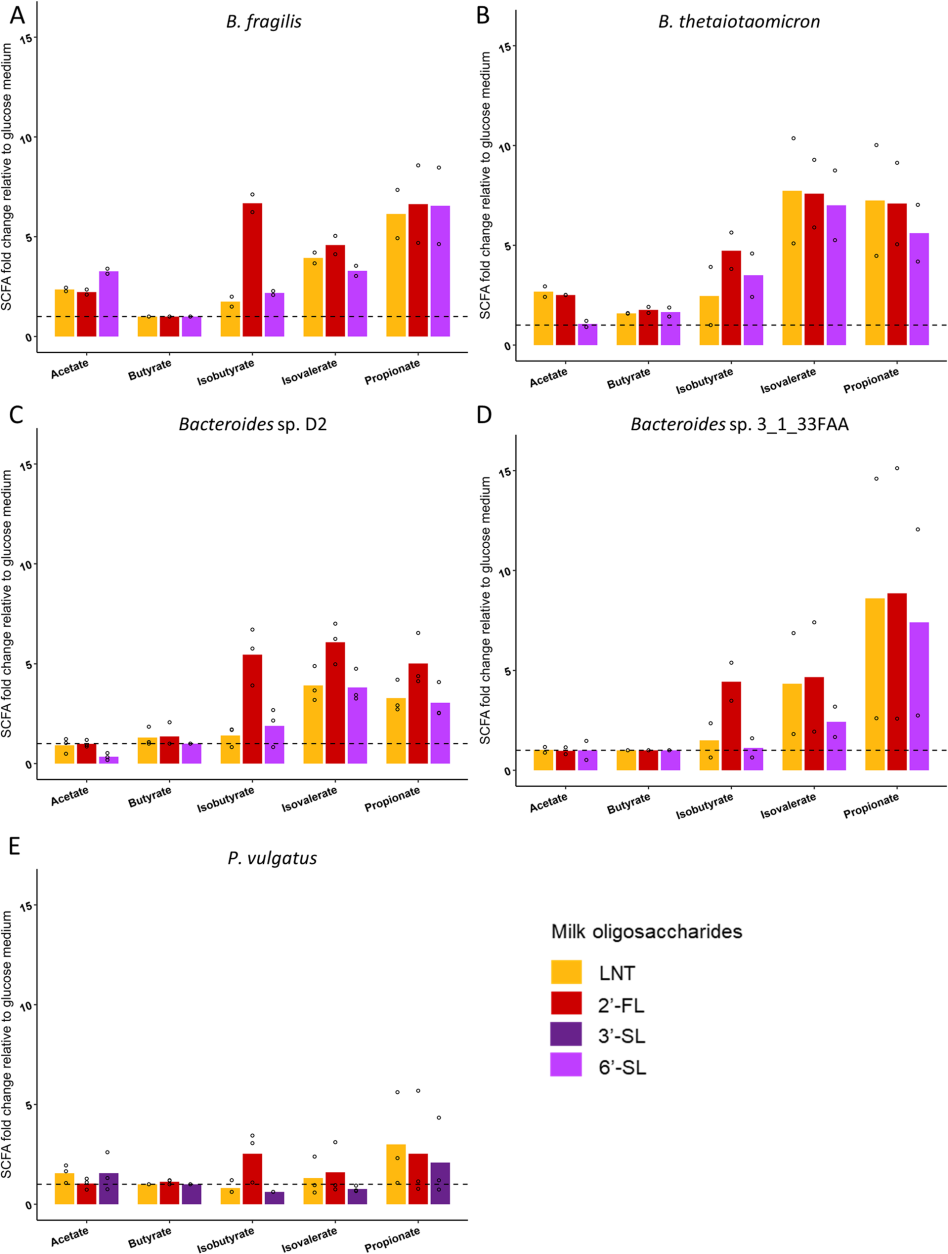
*In vitro* short chain fatty acids and branched chain fatty acids production by **A** B. fragilis **B** B. thetaiotaomicron **C** Bacteroides sp. D2 **D** Bacteroides sp. 3_1_33FAA (E) P. vulgatus cultivated in semi-defined media (mYCFA or mMRS) supplemented with 0.5% lacto-N-tetraose (LNT), 2’-fucosyllactose (2’-FL), 3’-sialyllactose (3’-SL) or 6’-sialyllactose (6’-SL). The concentrations were measured during the stationary phase. Values were calculated by subtracting the values from semi-defined media without bacterial strains. Data are expressed relative to the concentrations measured in the positive control (glucose). Barplots represent mean values and dots indicate values of each replicate (*n* = 2 or 3)

SCFA and BCFA concentrations in bacterial cultures with MOs were expressed relatively to the concentrations in the positive control i.e., with glucose, to appreciate the differences between MOs fermentation compared to a common carbon source (Fig. 7 and Supplementary Fig. 2). All bacteria growing with MOs produced propionate, isovalerate and isobutyrate, with concentrations ranging from similar to those observed with glucose to up to 10-fold higher, depending on the MOs and the bacteria. Interestingly, 2’-FL strongly stimulated the production of isobutyrate for all bacteria, with levels 3 to 6-fold higher than with glucose, while this induction was much more moderate or not observed with neutral (LNT) and sialylated (3’-SL or 6’-SL) oligosaccharides, with fold changes relative to glucose between 1 and 3 (Fig. 7). Taking into account the five strains growing with MOs, statistical analyses using the Kruskal-Wallis test revealed a significant difference between MOs (p-milk oligosaccharides < 0.05) for the production of isobutyrate (Table 2). *Post-hoc* analyses indicated significantly higher isobutyrate concentrations with 2’-FL than with LNT or 3’-SL or 6’-SL (*p* < 0.05) while no significant differences was detected between LNT and 3’-SL or 6’-SL. MOs degradation shows different patterns between the species. Fermentation of 2’-FL by *Bacteroides* sp. D2 strain produced higher amounts of isovalerate (6-fold higher *vs* 4-fold higher with LNT and 6’-SL) and propionate (5-fold higher *vs* 3-fold higher with LNT and 6’-SL) compared to LNT and 6’-SL (Fig. 7). While *P. vulgatus* strain produced less SCFAs and BCFAs compared to the other species, regardless of the MO structures, with a maximum of 3-fold higher production compared to glucose.

**Table 2:** Metabolites production between types of MOs regardless of the isolated strains. *P*-value was determined with the Kruskal-Wallis test, followed by post-hoc pairwise comparison with dunn’s test. For metabolites with *p*-value < 0.05, letters indicate mean significant differences between types of MOs

	Neutral (LNT) *n* = 12	Fucosylated (2’-FL) *n* = 12	Sialylated (3’-SL or 6’-SL) *n* = 12	Kruskal- Wallis *p*-value
	Mean ± SE	Mean ± SE	Mean ± SE	
GC-MS				
Acetate	1.63 ± 0.76	1.46 ± 0.7	1.36 ± 1.1	0.649
Butyrate	1.17 ± 0.31	1.25 ± 0.39	1.11 ± 0.27	0.411
Isobutyrate	1.51 ± 0.95^a^	4.64 ± 1.78^b^	1.76 ± 1.2^a^	< 0.001
Isovalerate	3.97 ± 2.69	4.72 ± 2.66	3.26 ± 2.29	0.258
Propionate	4.81 ± 4.06	5.42 ± 4.02	4.21 ± 3.45	0.518
NMR metabolites				
2-methylbutyrate	7.62 ± 3.39	8.61 ± 3.91	5.75 ± 2.84	0.138
Ethanol	1.46 ± 0.45^a^	0.49 ± 0.17^b^	1.13 ± 0.13^a^	< 0.001
Formate	2.22 ± 2.08	1.15 ± 0.5	1.23 ± 0.27	0.561
Fumarate	1.76 ± 2.41	1.62 ± 1.92	1.21 ± 1.11	0.577
Lactate	0.79 ± 0.52	0.68 ± 0.41	0.84 ± 0.72	0.928
Malic acid	2.75 ± 1.86	1.93 ± 1.8	2.4 ± 2.05	0.336
1,2-propanediol	2.24 ± 0.56^a^	41.47 ± 21.96^b^	1.83 ± 0.38^a^	< 0.001
Succinate	2.09 ± 1.36	1.64 ± 1.16	1.44 ± 0.99	0.367
Alanine	1.09 ± 0.16	1.08 ± 0.18	1.01 ± 0.21	0.603
Asparagine	1.32 ± 0.22	1.15 ± 0.15	1.18 ± 0.25	0.143
Aspartate	1.96 ± 2.88	1.94 ± 2.76	1.64 ± 1.97	0.996
Betaine	0.54 ± 0.07	0.49 ± 0.04	0.51 ± 0.05	0.414
Glutamate	1.72 ± 0.57	1.74 ± 0.47	1.44 ± 0.39	0.180
Glycine	1.39 ± 0.23	1.4 ± 0.12	1.63 ± 0.4	0.480
Isoleucine	1.06 ± 0.14	1.01 ± 0.19	0.98 ± 0.19	0.423
Leucine	1.02 ± 0.1	0.92 ± 0.11	0.99 ± 0.13	0.068
Lysine	1.08 ± 0.15	1.03 ± 0.11	1.01 ± 0.16	0.478
Methionine	1.36 ± 0.33	1.23 ± 0.25	1.26 ± 0.26	0.598
Phenylalanine	1.08 ± 0.09	0.99 ± 0.09	1.03 ± 0.12	0.109
Proline	2.3 ± 0.65	2.21 ± 0.58	1.78 ± 0.44	0.103
Pyroglutamate	1.11 ± 0.09	1 ± 0.09	1.04 ± 0.09	0.024
Tryptophane	1.01 ± 0.13	0.9 ± 0.15	0.99 ± 0.2	0.175
Tyrosine	1.17 ± 0.21	1.09 ± 0.19	1.08 ± 0.22	0.589
Valine	1.46 ± 0.21	1.36 ± 0.18	1.31 ± 0.19	0.260

### Metabolization of MOs resulted in a structure-dependent metabolome

We explored how MOs impacted the metabolic activity of the isolated bacteria able to grow with these carbohydrates by analyzing supernatant metabolome for metabolites other than SCFAs with NMR-based metabolomics. Supernatants generated during growth of *B. fragilis*, *B. thetaiotaomicron*, *Bacteroides* sp. D2, *Bacteroides* sp. 3_1_33FAA and *P. vulgatus* strains on LNT, 2’-FL, 3’-SL or 6’-SL or glucose allowed the identification of 36 different metabolites (Table S4). As observed for BCFA and SCFA, NMR-based metabolomes from supernatants of bacteria cultivated with MOs present a higher diversity than those cultivated with glucose (Table S6 and Supplementary Fig. 3).

In order to identify the metabolites whose production was the most associated with the structure of MOs, we performed a principal component analysis (PCA) on the species able to grow with MOs (Supplementary Fig. 4). The first two principal components of the PCA (Supplementary Fig. 4 A) accounted for 24 and 19% of the total variance and showed that the metabolome with MOs is distinguished from the control condition (glucose) according to the second component. Along the second component, metabolome from the glucose condition were characterized by a higher concentration in glucose, betaine and lactate (Supplementary Fig. 4B). No clustering according to the MO was observed.

**Fig. 8:**
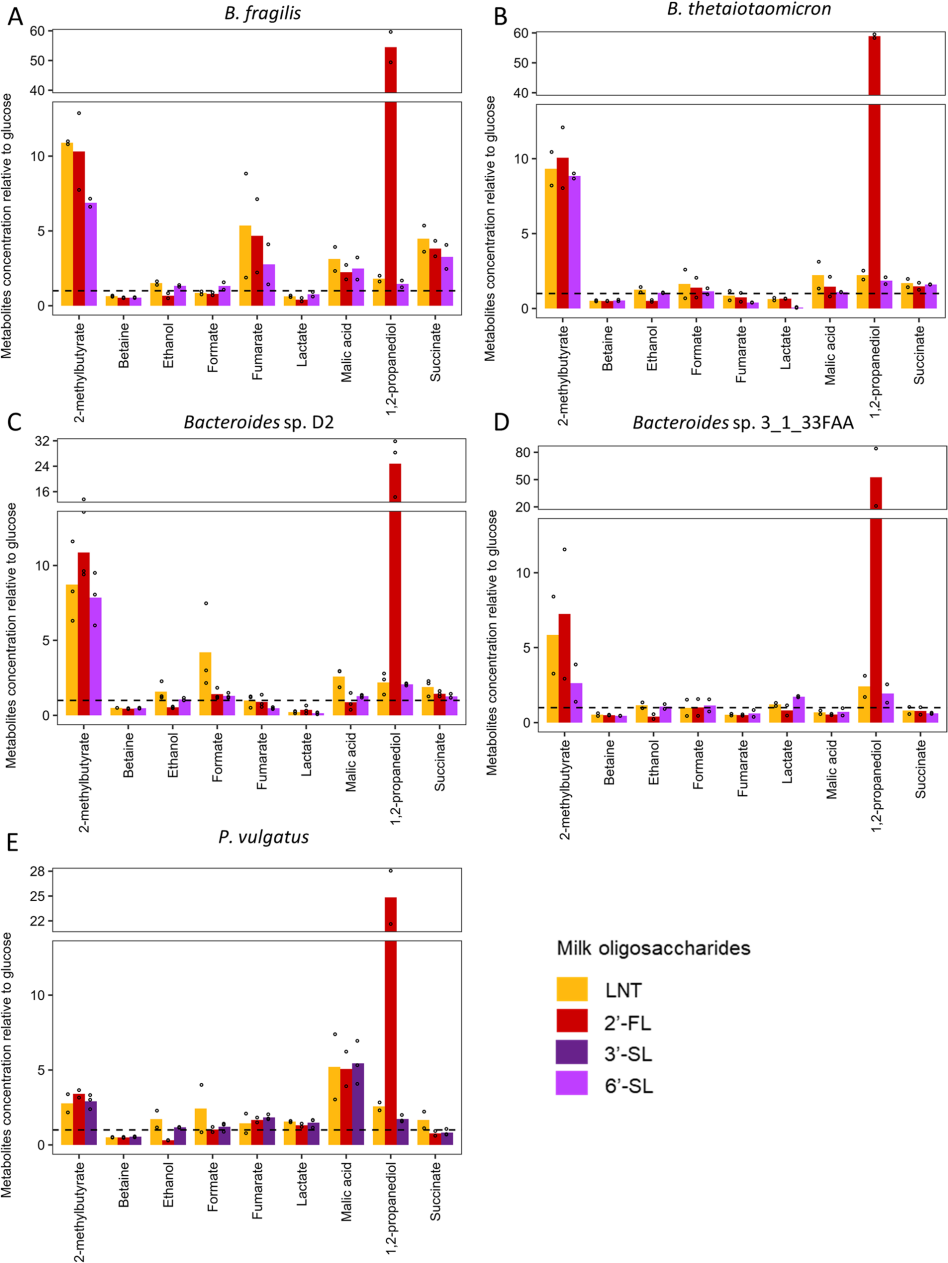
Relative concentration of bacterial metabolites after growth in semi-defined media (mYCFA) supplemented with either 0.5% lacto-N-tetraose (LNT), 2’-fucosyllactose (2’-FL), 3’-sialyllactose (3’-SL) or 6’-sialyllactose (6’-SL) of **A** B. fragilis **B** B. thetaiotaomicron **C** Bacteroides sp. D2 **D** Bacteroides sp. 3_1_33FAA **E** P. vulgatus. Data are expressed relative to the concentrations measured in the positive control (glucose). Barplots represent mean values and dots indicate values of each replicate (*n* = 2 or 3)

**Fig. 9:**
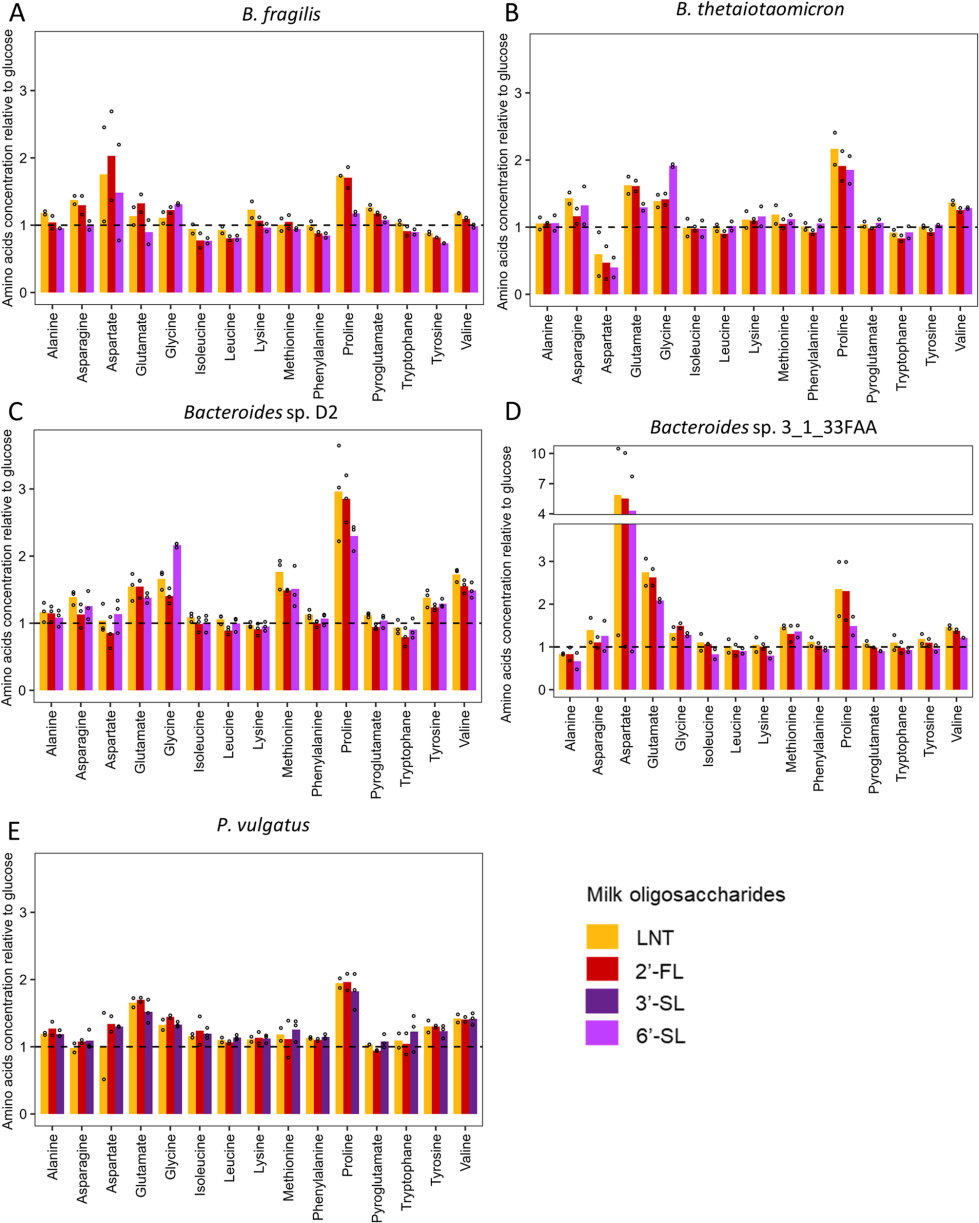
Relative concentration of amino acids after growth in semi-defined media (mYCFA) supplemented with 0.5% lacto-N-tetraose (LNT), 2’-fucosyllactose (2’-FL), 3’-sialyllactose (3’-SL) or 6’-sialyllactose (6’-SL) of **A** B. fragilis **B** B. thetaiotaomicron **C** Bacteroides sp. D2 **D** Bacteroides sp. 3_1_33FAA **E** P. vulgatus. Data are expressed relative to the concentrations measured in the positive control (glucose). Barplots represent mean values and dots indicate values of each replicate (*n* = 2 or 3)

Metabolite concentrations were expressed relative to the concentration of metabolites measured in the positive control i.e., bacteria grown with glucose, to appreciate the differences in MOs metabolization compared to a common carbon source (Figs. 8 and 9). Among BCFAs, 2-methylbutyrate, was markedly increased with MOs compared to glucose. Concentration in *B. fragilis*, *B. thetaiotaomicron* and *Bacteroides* sp. D2 were 9 to 11-fold higher compared to glucose when grown with 2’-FL and LNT, and 6 to 9-fold higher with 6’-SL, while this increase was more moderate for *Bacteroides* sp. 3_1_33FAA with 7, 5, and 2-fold higher concentrations compared to glucose with 2’-FL, LNT and 3’-SL, respectively. This increase was even lower with *P. vulgatus* strain where it averaged 3-fold higher compared to glucose regardless of the MO (Fig. 8). Betaine concentration was decreased with MOs compared to glucose in all strains, and in particular *Bacteroides* sp. D2 had a betaine concentration 2.2-fold lower compared to glucose for 2’-FL and 6’-SL and it was 2.2 lower for *Bacteroides* sp. 3_1_33FAA in 6’-SL. Ethanol production was always lower compared to glucose when cultivating with 2’-FL regardless of the bacteria (fold change ranging from 0.3 to 0.6). Statistical analyses using the Kruskal-Wallis test revealed a significant difference between the different milk oligosaccharides used for growth (p-milk oligosaccharides < 0.05) in ethanol production (Table 2). *Post-hoc* analyses indicated significantly lower ethanol production with 2’-FL compared to LNT or 3’-SL or 6’-SL (*p* < 0.05), while no significant differences were detected between LNT and 3’-SL or 6’-SL. LNT degradation by *Bacteroides* sp. D2 induced 4-fold higher formate levels compared to glucose while it was not increased with 6’-SL and 2’-FL. Fumarate concentrations were increased for all MOs compared to glucose (3 to 5-fold higher) in *B. fragilis*. Fermentation of 6’-SL by *B. thetaiotaomicron* and *Bacteroides* sp. D2 strains resulted in fumarate concentration 2 and 2.5-fold lower compared to glucose, respectively. Concentrations of malic acid in supernatants of *Bacteroides* sp. 3_1_33FAA grown with MOs were lower than with glucose (fold change ranging from 0.5 to 0.7), while they were 5-fold higher with all three MOs in *P. vulgatus* (Fig. 8). Succinate was produced in highest concentration in *B. fragilis* culture compared to the other bacteria (4.5-fold higher in LNT while 3.8 and 3-fold higher in 2’-FL and 6’-SL compared to glucose, respectively) (Fig. 8). Interestingly, 2’-FL greatly increased the relative concentration of the metabolite 1,2-propanediol, also known as propylene glycol, a precursor of propionate. The concentration of this metabolite increased more than 50-fold in *B. fragilis*, *B. thetaiotaomicron*, and *Bacteroides* sp. 3_1_33FAA strains, and 25-fold in *Bacteroides* sp. D2 and *P. vulgatus* after cultivation with 2’-FL while it was solely between 1.4 to 2.5-fold-higher with LNT and 3’-SL or 6’-SL (Fig. 8). Concentration of 1,2-propanediol was significantly higher with 2’-FL compared to LNT (*p* < 0.05) as well as 3’-SL or 6’-SL (*p* < 0.05) while no significant differences was detected between LNT and 3’-SL or 6’-SL (Table 2).

The concentrations of amino acids alanine, asparagine, lysine, phenylalanine, pyroglutamate, tryptophane and tyrosine, were similar compared to glucose or between MO structures (Fig. 9). Glutamate displayed slight increases with all MOs compared to glucose for *B. thetaiotaomicron*, *Bacteroides* sp. D2, *Bacteroides* sp. 3_1_33FAA and *P. vulgatus* strains (fold change between 1.5 and 2.7). Similarly, for all bacteria, proline displayed slight increases with all MOs compared to glucose (fold change between 1.7 and 2.9). Aspartate concentrations were differentially impacted between species: no difference in its production between glucose and MOs were observed for *Bacteroides* sp. D2 and *P. vulgatus* while a moderate (1.5 to 2-fold) and high (4.3 to 6-fold) increase was observed in *B. fragilis* and *Bacteroides* sp. 3_1_33FAA respectively. Conversely, aspartate concentration in *B. thetaiotaomicron* was 2 to 2.5-fold lower compared to glucose with 2’-FL and 6’-SL respectively (Fig. 9). Finally, the concentration of branched amino acids leucine and isoleucine was not modulated or slightly decreased with MOs (0.6 to 1-fold higher compared to glucose) while valine was slightly increased (1.2 to 1.5-fold higher compared to glucose) for all bacteria except *B. fragilis* (Fig. 9).

### Identification of glycoside hydrolases in the strain genomes

Except for *B. uniformis* strain whose DNA extraction was unsuccessful, the genome of the selected strains able to grow or not with MOs, was sequenced (Table 3). Affiliation using whole genome is in agreement with 16 S taxonomic affiliation except for two bacteria: *Bacteroides* sp. D2 and *Bacteroides* sp. 3_1_33FAA. At the strain level, *H. hathewayi* and *P. vulgatus* are the only bacteria showing the same affiliation for 16 S and whole genome sequencing.

**Table 3:** Genome characteristics of the 8 intestinal commensal strains isolated from suckling piglets and rabbits

ID	Genome length (bp)	GC content (%)	Number of contigs	Specie predicted by genome sequencing (Reference genome)	Specie predicted by 16 S ribosomal gene sequencing (Reference sequences)	Average nucleotide identity ANI	Tetra-nucleotide correlation	Jspecies Z score
Isolate_71	5 389 741	43.5	3	*Bacteroides fragilis* str. I1345	*Bacteroides fragilis* NCTC 9343	98.50	0.99929	0.99929
Isolate_128	6 514 178	43.1	2	*Bacteroides thetaiotaomicron* 1_1_6	*Bacteroides thetaiotaomicron* VPI-5482	98.15	0.99936	0.99936
Isolate_130	6 762 123	41.6	1	*Bacteroides* sp. D2	*NQMG_s* KFT8	94.86	0.99916	0.99907
Isolate_55	6 094 260	41.9	12	*Bacteroides* sp. 3_1_33FAA	*Phocaeicola dorei* DSM 17,855	97.71	0.99864	0.99864
Isolate_30	6 398 705	48.4	2	*Hungatella hathewayi* DSM 13,479	*Hungatella hathewayi* DSM 13,479	98.99	0.99948	0.99948
Isolate_180	5 119 265	42.4	4	*Phocaeicola vulgatus* ATCC 8482	*Phocaeicola vulgatus* ATCC 8482	97.73	0.99916	0.99916
Isolate_183	2 233 298	38.0	3	*Lactobacillus amylovorous* GRL 1112	*Lactobacillus amylovorous* DSM 20,531	98.07	0.99929	0.9977
Isolate_190	2 246 820	46.6	2	*Limosilactobacillus mucosae* DSM 13,345	*Limosilactobacillus mucosae* S32	96.87	0.99866	0.99866

To investigate the putative enzymes implicated in the degradation of LNT, 2’-FL, 3’-SL and 6’-SL, the 8 genomes were annotated against the CAZy database. We focused our investigation on GH families targeting the linkages found within the MOs: β-galactosidase, Lacto-N-biosidase, α−1,2-fucosidase, α−2,3-sialidase and α−2,6-sialidase (Fig. 2). In the genomes of *B. fragilis*, *B. thetaiotaomicron* and *Bacteroides* sp. D2 species, several putative Lacto-N-biosidases from the family GH20 were identified, while the genomes of *Bacteroides* sp. 3_1_33FAA and *P. vulgatus* contain putative Lacto-N-biosidases of both the GH20 and GH136 families (Table 4). Lacto-N-biosidases might be implicated in the hydrolysis of the LNT into two components, lacto-N-biose (LNB) (Gal(β1–3)GlcNAc) and lactose (Gal(β1–4)Glc). *B. fragilis*, whose consumption of 6’-SL was particularly efficient, possesses 6 putative sialidases (GH33) which target the NeuAc linked in α2-3- or α2-6- resulting in the release of NeuAc and lactose (Gal(β1–4)Glc). These putative sialidases were also found in the genomes of *B. thetaiotaomicron*, *Bacteroides* sp. D2, *Bacteroides* sp. 3_1_33FAA and *P. vulgatus* (2 to 4 gene copies) (Table 4). Putative α−1,2-L-fucosidases (GH95), which target the fucose linked in α1-2- to release the Fuc and lactose, are particularly abundant in the *Bacteroides* sp. D2 (10 GH95) and *Bacteroides* sp. 3_1_33FAA (8 GH95) genomes while *B. fragilis* and *B. thetaiotaomicron* genomes include 4 and 6 GH95, respectively. *P. vulgatus* genome includes 3 putative α−1,2-L-fucosidases (GH95) (Table 4). We also identified a total of 22, 45 and 36 putative β-galactosidases belonging to the families GH2 and GH35 in the genomes of *B. fragilis*, *B. thetaiotaomicron* and *Bacteroides* sp. D2, respectively (Table 4). These enzymes allow the bacteria to hydrolyze lactose (Galβ1-4Glc), issued from the four MOs studied, once it has been freed from the other monosaccharides of the MOs with the mechanisms described above. β-galactosidases are also predicted to hydrolyze the Gal from the lacto-N-biose (Gal(β1–3)GlcNAc(β1–3)) resulting from the LNT hydrolysis by lacto-N-biosidases. *Bacteroides* sp. 3_1_33FAA and *P. vulgatus* genomes include 25 and 22 putative β-galactosidases respectively from the GH2, GH35 and GH42 families (Table 4). Consequently, the five bacteria that were able to grow with MOs have indeed a large repertoire of GHs in their genome, with copy numbers similar to those we found in CAZy database (Table 4).

Surprisingly, *H. hathewayi*, which was not able to consume any of the MOs studied, has many GHs in its genome (Table 4). Indeed, it possesses 24 putative β-galactosidases (GH1, GH2, GH35 and GH42) along with 4 putative lacto-N-biosidases (GH20 and GH136), 3 putative lacto-N-biose phosphorylases (GH112) and 2 putative sialidases (GH33) which implies that it may be capable of metabolizing LNT and 6’-SL. However, its genome does not include any putative α−1,2-L-fucosidases, which explains its inability to consume 2’-FL. *L. amylovorus* possesses solely 2 putative β-galactosidases which is consistent with its incapacity to metabolize the MOs. Similarly, *L. mucosae* strain possesses solely 2 putative β-galactosidases along with 5 putative β−1,4-galactosidases (Table 4). However, since they do not possess the enzymes to hydrolyze the lacto-N-biose, the fucose or the sialic acid, they cannot use MOs as a substrate.

**Table 4:** Copy numbers of MOs-targeted glycoside hydrolase families in strains isolated from suckling rabbits and piglets. * Numbers in bold are the copy number found in the whole genome annotations **Numbers in italic are the predicted copy number from the CAZy database based on the 16 S taxonomic affiliation. ***NA indicates that the 16 S taxonomic affiliation were not found in the CAZy database. GH = Glycoside hydrolase

GH family	Enzyme activity	Gene copy number							
		B. fragilis (Isolate_71)	B. thetaiotaomicron (Isolate_128)	Bacteroides sp. D2 (Isolate_130)	Bacteroides sp. 3_1_33FAA (Isolate_55)	H. hathewayi (Isolate_30)	*P*. vulgatus (Isolate_180)	L. amylovorous (Isolate_183)	L. mucosae (Isolate_190)
GH1	β−1,4-galactosidase	0* 0**	**0** *0*	**0** NA***	**0** *0*	**8** *8*	**0** *0*	**0** *6*	**5** *NA*
GH2	β-galactosidase	**17** *15*	**32** *31*	**36** *NA*	**23** *27*	**13** *13*	**20** *25*	**1** *1*	**1** *NA*
GH20	Lacto-N-biosidase	**12** *12*	**13** *14*	**15** *NA*	**11** *10*	**2** *2*	**10** *9*	**0** *0*	**0** *NA*
GH33	α−2,3-sialidase; α−2,6-sialidase	**6** *3*	**2** *2*	**4** *NA*	**3** *2*	**2** *2*	**3** *2*	**0** *0*	**0** *NA*
GH35	β-galactosidase	**5** *4*	**3** *3*	**7** *NA*	**1** *1*	**1** *1*	**1** *1*	**0** *0*	**0** *NA*
GH42	β-galactosidase	**0** *0*	**1** *1*	**2** *NA*	**1** *1*	**2** *2*	**1** *1*	**1** *1*	**1** *NA*
GH95	α−1,2-L-fucosidase	**4** *3*	**6** *5*	**10** *NA*	**8** *4*	**0** *0*	**3** *4*	**0** *0*	**0** *NA*
GH112	Lacto-N-biose phosphorylase	**0** *0*	**0** *0*	**0** *NA*	**0** *0*	**3** *3*	**0** *0*	**0** *0*	**0** *NA*
GH136	Lacto-N-biosidase	**0** *0*	**0** *0*	**0** *NA*	**1** *1*	**2** *2*	**2** *1*	**0** *0*	**0** *NA*

## Discussion

Milk oligosaccharides have gathered significant attention in the scientific community due to their impact on the gut microbiota of neonates. Present in all mammalian milks, MOs display considerable structural variation across species. Emerging evidence indicates a correlation between the structure and functions of MOs, suggesting that the molecular assembly of MOs can yield diverse effects on the gut microbiota. However, studies investigating the effects of individual MOs on isolated commensal bacteria are scarce [22, 24] and focus mainly on human isolated *Bifidobacteria* [23–28], even though these bacteria are less prevalent in the gut microbiome of young individuals from other mammalian species such as pigs and rabbits [29, 30]. In this study, we used culture-dependent methods to isolate newly intestinal commensal bacteria using feces of suckling piglets and cecum content from suckling rabbits and evaluate the impact of individual MOs. Through anaerobic culture methods, we successfully obtained a collection of 240 distinct bacterial isolates.

In rabbits, 36% of the isolates belonged to the facultative anaerobic *Enterobacteriaceae* family, which was also prevalent in the study of Gouet and Fonty [55] in 21 and 25 days old rabbits. In our study, the use of antibiotics in the culture media reduced the prevalence of this family in subsequent cultures. However, this strategy may have inhibited the growth of other bacteria sensitive to antibiotics. In accordance with previous studies [55], we did not isolate any *Lactobacillaceae* from rabbit cecal contents, while *Bacteroidaceae* emerged as the dominant isolated and cultivated family, comprising 48% of the isolates. Regarding microbiota composition obtained previously by 16 S rRNA gene analysis of cecal microbiota, Paës et al. [56] and Beaumont et al. [57] reported that, in 25 days old rabbits, *Bacteroidaceae*,* Lachnospiraceae* and *Ruminococcaceae* are the most abundant family. Consequently, the main taxa from the microbiota of rabbits are represented in our isolates. This validates our culture-based approach and confirm that our collection is representative of the rabbit intestinal microbiota. In piglets, the primary phyla isolated in feces were *Bacillota* (formerly *Firmicutes*) and *Pseudomonadota* (formerly *Proteobacteria*) in line with other culture-dependent studies [58, 59]. The main families isolated in our study were *Enterobacteriaceae* (37%), *Lactobacillaceae* (16%), *Streptococcaceae* (15%) and *Bacteroidaceae* (11%), which are not the most dominant on the fecal microbiota of piglets [60], pointing out that culture-dependent isolated families are overrepresented in comparison to the original microbial community in this host. At the genera level, the ones found as dominant– 37% of *Escherichia*, 15% of *Lactobacillus* (including *Ligilactobacillus* and *Limosilactobacillus*, in accordance with prior taxonomic classifications) and 15% of *Streptococcus*– are the same ones found in a previous culture-dependent study [58]. Including *Phocaeicola*, previously classified as *Bacteroides* [61], the genus *Bacteroides* becomes the fourth most abundant isolated genera. Similar observations were made by Wang et al. [58], who detected *Bacteroides* in nursing piglets and concluded that it could possibly be linked to the consumption of milk-derived proteins, lactose and galactose. In general, culture-dependent methods used in this study have been effective for recovering bacteria for subsequent *in vitro* experiments; however, they have limits and only a subset of microorganisms is normally identified as several species in the microbiota may not be cultivable with the current methods. For future studies, it may be interesting to develop new media and culture conditions to capture what is not currently cultivable.

Among the 240 isolated bacteria, we selected two *Bacteroidaceae* from the pig isolates collection, along with four *Bacteroidaceae* and one *Lachnospiraceae* from rabbit gut isolates as these species possess a putative range of GHs predicted to degrade MOs. We also included two *Lactobacillaceae* due to their probiotic potential. We cultivated them with LNT, 2’-FL, and 3’-SL or 6’-SL for piglets and rabbits respectively as sole carbon source.

Our findings indicate that four *Bacteroides* and one *Phocaeicola* strains (formerly classified as *Bacteroides* [61]) isolated from suckling rabbits and piglets were able to consume MOs. The ability of *Bacteroides* species to metabolize MOs has been described in previous research [31, 32, 62, 63]. *Bacteroides* are known for their capacity to utilize complex carbohydrates, particularly mucin glycans, thanks to the presence of polysaccharide utilization loci (PUL) in their genomes. Notably, Marcobal et al. [40] showed that *B. thetaiotaomicron* employs PUL associated with mucin utilization when grown on MOs. The only *Bacteroides* strain in our study that could not grow on MOs is to *B. uniformis*, consistent with previous findings [64, 65]. Our results indicate that ability to utilize MOs are species-dependent, which aligns with the six *Bacteroides* species observations from Marcobal et al. [40].

Interestingly, the growth and MO consumption patterns of the strains appeared to depend on the specific structure of the MOs. While 2’-FL resulted in an important growth across all species, 3’-SL or 6’-SL and LNT were more selective. *B. fragilis* responded to the three types of MOs with robust growth. This finding corroborates with the results found by Marcobal et al. [31], who reported high consumption rates of nearly all MO structures by *B. fragilis* from a mixture of 14 structures. *B. thetaiotaomicron* exhibited robust growth with 2’-FL but showed reduced capacity to consume 6’-SL, contrasting with the results obtained by Chia et al. [63], who observed an important utilization of 6’-SL by *B. thetaiotaomicron* with less than 50% of 6’-SL left in the supernatant after 48 h of degradation of a mix of MOs. They also reported the utilization of monosaccharides belonging to LNT and 2’-FL by *B. thetaiotaomicron* throughout the degradation of a mix of MOs with an absence of residues of GlcNAc and Fuc. In our study, we also did not find GlcNAc in the supernatant after growth of *B. thetaiotaomicron* with LNT. However, after growth with 2’-FL we observed considerable levels of Fuc indicating that *B. thetaiotaomicron* is unable to efficiently metabolize this monosaccharide in our experimental conditions. These divergences in results could be attributed to differences in the strains used in the studies (VPI-5428 in Chia et al. [63] vs. 1_1_6 in our study), and the fact that they cultured the bacteria with a mixture of MOs, while we used single MOs. Similarly, our results with *Bacteroides* sp. D2 demonstrated efficient growth with both 2’-FL and LNT but poor utilization of 6’-SL. *Bacteroides* sp. 3_1_33FAA exhibited substantial growth with all three MOs, with minimal residues of MOs remaining in the supernatant during the stationary phase. Kijner, Cher, Yassour [66] demonstrated the ability of *P. dorei*, a species closely related to *Bacteroides* sp. 3_1_33FAA to grow with LNT, 2’-FL and 6’-SL. *P. vulgatus* grew with all three MOs, although growth was notably less pronounced with 3’-SL. In our study, *P. vulgatus* showed a preference for 2’-FL, consistent with findings from Marcobal et al. [31]. Their study found that the fucosylated MOs were the most consumed by *P. vulgatus* ATCC8482, as they were present in the lowest amounts in the supernatant after growth with a mixture of MOs.

The five strains metabolized the MOs into various metabolites, with production patterns depending on the structure of the MOs. Specifically, 2’-FL stimulated the production of propionate and the BCFAs isovalerate, isobutyrate and 2-methylbutyrate. We expected a reduction of leucine and isoleucine levels, precursors for BCFAs present in the YCFA media, possibly due to their utilization for the production of BCFAs [67], however they were not reduced when bacteria were grown with MOs compared to glucose. Several bacterial species have already shown an increase in the production of branched chain fatty acids in a peptone yeast medium compared to a peptone yeast medium with glucose [68]. The presence of carbohydrates was hypothesized to inhibit the proteolytic activity of the bacteria. MOs being less accessible than glucose, degradation of these carbohydrates is longer and proteolytic activity might consequently be higher with MOs. One notable difference in metabolite production based on MOs structure was the pronounced increase in 1,2-propanediol with 2’-FL in all bacteria tested. This metabolite, also known as propyleneglycol, has previously been identified as a product of fucose metabolism in *Bifidobacterium* species and *Akkermansia muciniphila* [13, 15, 69, 70]. 1,2-propanediol is an intermediary metabolite derived from the microbial degradation of deoxyhexose sugars like fucose and is involved in the production of propionate [71]. However, studies suggest that *Bacteroides* species typically produce 1,2-propanediol as their final product [72]. We hypothesize that, following hydrolysis of 2’-FL, *Bacteroides* species might utilize the glucose and galactose residues to produce propionate while converting fucose into 1,2-propanediol.

The analysis of the genome indicates that the five species possess a repertoire of GHs, aimed at all MOs structures investigated. All strains possess lacto-N-biosidase, to cleave the LNT in lacto-N-biose (Gal(β1–3)GlcNAc) and lactose (Gal(β1–4)Glc), and β-glucosidase to cleave the lactose, however they lack the lacto-N-biose phosphorylase required to cleave the lacto-N-biose. This could explain the low quantities of N-acetylglucosamine detected in the supernatant at the end of the culture, this monomer not being released from the lacto-N-biose. Among the *Bacteroidetes* studied, all had several GH33 in their genome. GH33 which acts by separating the lactose and the sialic acid residue was found in the genome of *B. thetaiotaomicron* in two different PUL suggesting that *Bacteroidetes* species utilize different mechanisms to metabolize MOs [65]. Despite the low consumption of 6’-SL by *B. thetaiotaomicron*, we found 6-fold higher NeuAc than in the uninoculated medium. This can be explained by the lack of the enzymes specialized in the metabolization of sialic acid [73]. On the contrary, *B. fragilis* had the same amount of NeuAc left in the medium as *B. thetaiotaomicron* for a higher consumption of MOs, which suggests that the bacterium can metabolize NeuAc. Marcobal et al. [40] already demonstrated that *B. fragilis* has an extensive set of genes dedicated to sialylated MOs including genes to catabolize NeuAc after cleavage from the 6’-SL [40].

None of the *Lactobacillaceae* strains tested were able to grow on MOs, which is consistent with the absence of GHs in their genome, and prior studies showing little (2’-FL, LNnT) to no growth (3’-SL) of bacteria from this family with MOs [32, 74]. Thongaram et al. [74] reported moderate growth of several *Lactobacillus* strains on LNnT, yet they had the ability to consume monosaccharide components of LNnT, which are similar to those of LNT (Glc, Gal and GlcNAc) [74, 75]. Therefore, *in vivo*, *Lactobacillaceae* could take advantage of monosaccharides released from MOs by other bacterial species present in the gut microbiota. Similarly, *H. hathewayi*, was not efficient at utilizing MOs. Although no studies have directly tested this species with MOs, our investigation did reveal the presence of multiple GHs in its genome. The absence of degradation observed could be due to the absence of extracellular GHs and/or specialized transporters to capture, and import MOs to be metabolized intracellularly [76].

## Conclusion

We demonstrated the ability of newly isolated *Bacteroides* and *Phocaeicola* commensal bacteria, from suckling piglets and rabbits, to selectively consume MOs, with growth and metabolic profiles dependent on the structure of these molecules. Notably, growth with 2’-FL resulted in higher bacterial biomass for all bacteria and 1,2-propanediol and isobutyrate productions were different compared to the other MOs. Genomic analyses revealed an abundance of GHs in the genome of these bacteria, suggesting a genetic adaptation that facilitates the utilization of MOs. The ability of these commensal bacteria to thrive in the presence of MOs suggests that these compounds could be used to modulate the gut microbiota. Furthermore, the increased production of certain metabolites after MOs degradation could impact host health. The study of the effects of these metabolites on the intestinal barrier function could provide a better understanding of the role of MOs in health. One of the limits of our study is the actual state of the art on sequences assembly as well as the actual thresholds defined for species identification, which evolves rapidly inducing new taxonomical reorganizations. This limit, which is common to all the studies using microbial genomes, is a view of the complexity of the bacteria communities. Besides, a deeper analysis of the culture conditions increasing MO degradation has not been done to our knowledge up to day, which could indicate that the actual techniques used to study MO degradation could benefice from other culture methods that the ones employed until now. Furthermore, our study highlights the need of future in-depth analysis of genome sequences associated with bacterial genetic engineering which will be key to validate enzymatic capacity of bacteria to degrade MOs and even to identify new GH and the pathways involved. In addition, in-depth analysis of changes in metabolites during the degradation of MOs could reveal specific metabolic pathways and key intermediates in the degradation of MOs that are unknown until now.

## Supplementary Information

Supplementary Material 1.

Supplementary Material 2.

Supplementary Material 3.

Supplementary Material 4.

## Data Availability

All data generated or analyzed during this study are included in this published article [and its supplementary information files]. Sequences are available at European Nucleotide Archive (ENA) repository under the reference PRJEB86776. Isolated strains used and/or analyzed during the current study are available from the corresponding author on reasonable request.

## Abbreviations

2’-FL: 2’-fucosyllactose
3’-SL: 3’-sialyllactose
6’-SL: 6’-sialyllactose
ANI: Average nucleotide identity
BBE: Bacteroides bile esculin agar
BCFA: Branched chain fatty acid
BHIS: Brain heart infusion
CAZyme: Carbohydrate active enzyme
CG: Gas chromatograph
Fuc: Fucose
Gal: Galactose
GAM: Gifu Anaerobic Medium
GH: Glycoside hydrolase
Glc: Glucose
GlcNAc: N-acetylglucosamine
HMOs: Human milk oligosaccharides
^1^H-NMR: ^1^H-Nuclear magnetic resonance
LKV: Laked Brucella Blood Agar
LNB: Lacto-N-biose
LNT: Lacto-N-tetraose
MO: Milk oligosaccharides
MRS: Man Rogosa and Sharpe
NeuAc: N-acetylneuraminic acid
OD: Optical density
PUL: Polysaccharide utilization loci
RCM: Reinforced Clostridial medium
SCFA: Short chain fatty acid
YCFA: Yeast extract casitone and fatty acid broth
