# Supplementary material for: Structure-dependent degradation of milk oligosaccharides by newly isolated intestinal commensal bacterial strains from suckling piglets and rabbits
Source: BMC Microbiol. 2025 Aug 16;25:513. doi: 10.1186/s12866-025-04205-y (PMC12357397; doi:10.1186/s12866-025-04205-y)
Supplement: Supplementary file 1 — Supplementary Material 1. [file 12866_2025_4205_MOESM1_ESM.docx]

Table S1: Characteristics of the individuals sampled for the experiments

| **Individual** | **Pig 1** | **Pig 2** | **Pig 3** | **Rabbit 1** | **Rabbit 2** | **Rabbit 3** |
| --- | --- | --- | --- | --- | --- | --- |
| **Genetic** | Piétrain x (Large White x Landrace) | | | INRAE 1777 | | |
| **Sex** | Female | Male | Male |  |  |  |
| **Age at sampling** | 28 days | | | 16 days | 23 days | |
| **Body weight (Kg)** | 9.36 | 8.94 | 8.28 | 0.315 | 0.438 | 0.428 |

Table S2: List of primers used to amplify the whole 16S rRNA gene

| Host | Direction | Sequence (5’-3’) |
| --- | --- | --- |
| Rabbit | Forward | ACGGCTACCTTGTTACGACTT |
|  | Reverse | AGAGTTTGATCCTGGCTCAG |
| Pig | Forward | AGAGTTTGATCATGGCTCA |
|  | Reverse | TACGGTTACCTTGTTACGACTT |

Table S4: Metabolites identified by ^1^H-NMR metabolomics in bacterial culture supernatants

|  | Metabolites | δH (ppm) |
| --- | --- | --- |
| 1 | 2-methylburyrate | 0.86* (m), 1.05 (d), 1.39 (m), 1.50 (m), 2.20 (m) |
| 2 | Isovalerate | 0.92* (d), 1.98 (m), 2.12 (d) |
| 3 | Leucine | 0.97* (m), 1.72 (m), 3.74 (m) |
| 4 | Valine | 0.99 (d), 1.05* (d), 2.28 (m), 3.62 (d) |
| 5 | Isoleucine | 0.94 (m), 1.15* (d), 1.26 (m), 1.47 (m), 1.98 (m), 3.68 (d) |
| 6 | Propionate | 1.06* (m), 2.19 (m) |
| 7 | 1,2-propanediol | 1.15* (d), 3.45 (m), 3.55 (m), 3.89 (m) |
| 8 | Ethanol | 1.19* (m) |
| 9 | Fucose | 1.21* (d), 1.25 (d), 3.36 (s), 3.46 (m), 3.65 (m), 3.79 (m), 4.20 (m), 4.56 (d), 4.80 (s), 5.21 (d) |
| 10 | Alanine | 1.49* (d), 3.79 (m) |
| 12 | Acetate | 1.92* (s) |
| 11 | N-acetylneuraminic acid | 1.83 (m), 2.06* (d), 2.22 (m), 3.53 (d), 3.62 (m), 3.77 (m), 3.84 (d), 3.86 (d), 3.92 (m), 3.98 (m) |
| 13 | Glutamate | 2.07 (m), 2.36* (m), 3.77 (m) |
| 14 | Succinate | 2.41* (s) |
| 15 | Pyroglutamate | 2.041 (m), 2.41 (m), 2.51* (m), 4.18 (m) |
| 16 | Methionine | 2.14 (s), 2.20 (m), 2.65* (m), 3.86 (m) |
| 17 | 3’-sialyllactose | 1.18 (m), 1.92 (s), 2.04 (s), 2.76* (m), 3.30 (m), 3.57 (m), 4.11 (m), 4.53 (d), 4.68 (d), 4.8 (s), 5.24 (d) |
| 18 | Aspartate | 2.69 (m), 2.80* (d), 2.83 (d), 3.90 (m) |
| 19 | Asparagine | 2.86 (m), 2.89 (m), 2.94* (m), 2.97 (m) |
| 20 | Lysine | 1.49 (m), 1.74 (m), 1.92 (m), 3.03* (m), 3.76 (m) |
| 21 | Betaine | 3.27* (s) |
| 22 | Proline | 3.35* (m), 4.13 (m) |
| 23 | Glucose | 3.25 (m), 3.41 (m), 3.48* (m), 3.74 (m), 3.79 (m), 3.84 (m), 3.89 (m), 3.91 (m), 4.65 (d), 4.8 (s), 5.24 (d) |
| 24 | Glycine | 3.57* (s) |
| 25 | Lactate | 1.33 (d), 4.12* (m) |
| 26 | Malic acid | 2.66 (d), 2.69 (d), 4.29* (d), 4.31 (d) |
| 27 | 6’-sialyllactose | 1.75 (m), 1.92 (s), 2.04 (s), 2.72 (m), 3.32 (m), 3.53 (m), 3.8 (m), 4.44* (d), 4.69 (d), 5.24 (d) |
| 28 | Lacto-N-tetraose | 2.04 (s), 2.14 (s), 3.3 (m), 3.77 (m), 4.17 (d), 4.46* (d), 4.67 (d), 5.24 (d) |
| 29 | N-acetylglucosamine | 1.92 (s), 1.95 (s), 2.06 (s), 2.14 (m), 3.36 (s), 3.48 (m), 3.56 (m), 3.68 (m), 3.78 (m), 3.87 (m), 4.73 (d), 5.24* (d) |
| 30 | Galactose | 4.59 (d), 5.27* (d) |
| 31 | 2’-fucosyllactose | 1.24 (d), 1.92 (s), 3.32 (m), 3.49 (m), 3.58 (m), 3.79 (m), 4.24 (m), 4.54 (d), 4.65 (d), 5.25 (d), 5.32* (s) |
| 32 | Fumarate | 6.52* (s) |
| 33 | Tyrosine | 3.068 (m), 3.20 (m), 3.95 (m), 6.91* (d), 7.20 (d) |
| 34 | Phenylalanine | 3.14 (m), 3.28 (m), 4 (m), 7.38 (m), 7.43* (m) |
| 35 | Tryptophane | 7.2 (d), 7.28 (m), 7.3 (s), 7.55 (d), 7.74* (d) |
| 36 | Formate | 8.46* (s) |

*: Indicates the peak used for quantification based on the corresponding bucket intensity (not overlapping with peaks from other metabolites). Multiplicity of signals is indicated within brackets: s, singlet; d, doublet and m, multiplet


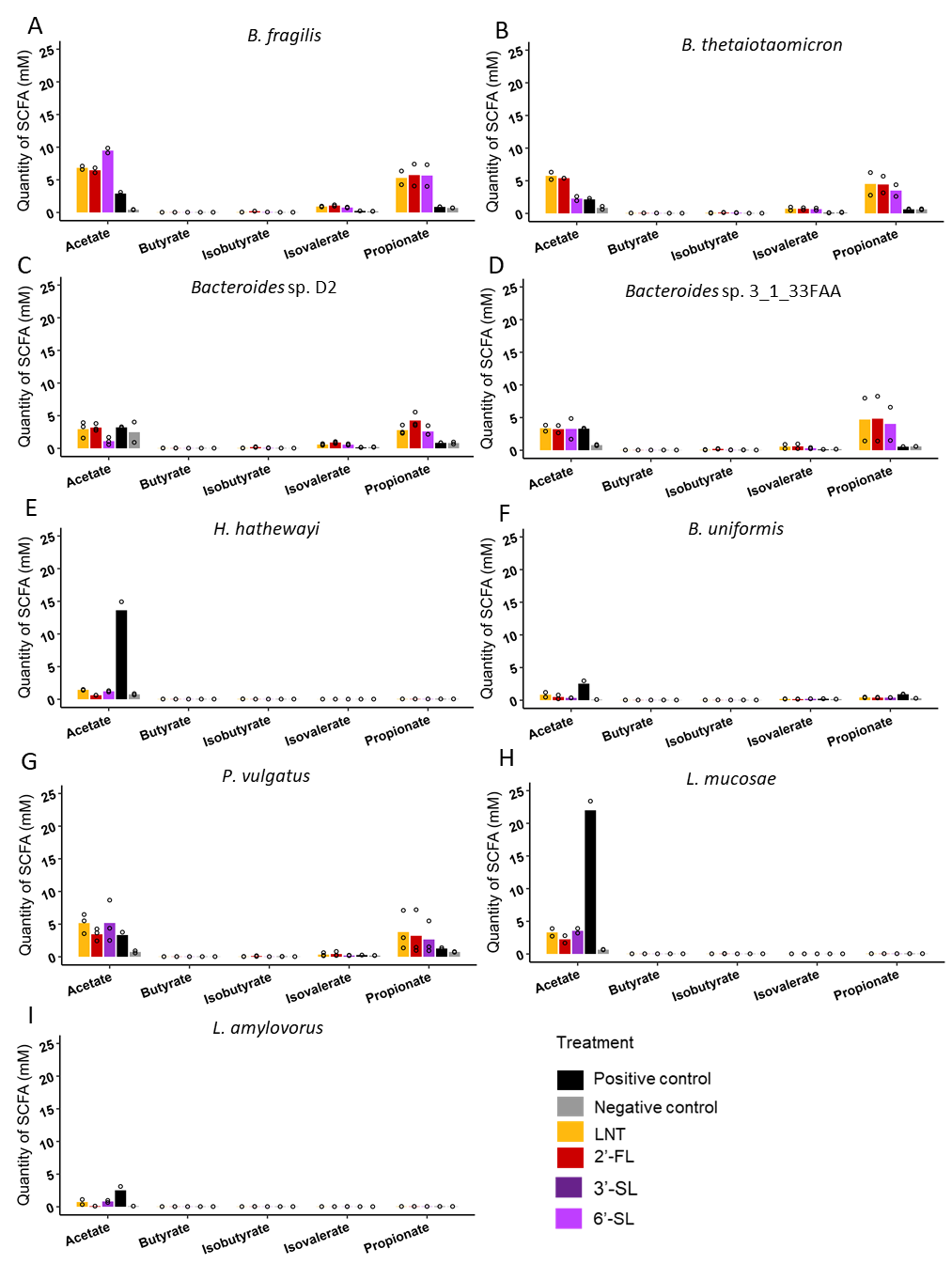


Supplementary Figure 1: In vitro short chain fatty acids and branched chain fatty acids production from (A) B. fragilis (B) B. thetaiotaomicron (C) Bacteroides sp. D2 (D) Bacteroides sp. 3_1_33FAA (E) H. hathewayi (F) B. uniformis (G) P. vulgatus (H) L. mucosae (I) L. amylovorus cultivated in semi-defined media (mYCFA or mMRS) supplemented with 0.5% glucose (positive control), lacto-N-Tetraose (LNT), 2'-fucosyllactose (2’-FL), 3’-sialyllactose (3’-SL) or 6'-sialyllactose (6’-SL) or no carbon source (negative control). The concentrations were measured during the stationary phase. Values were calculated by subtracting the values from semi-defined media without bacterial strains. Data are presented as absolute concentration; histograms represent mean values and dots represent the values of each replicate (n = 2 or 3).


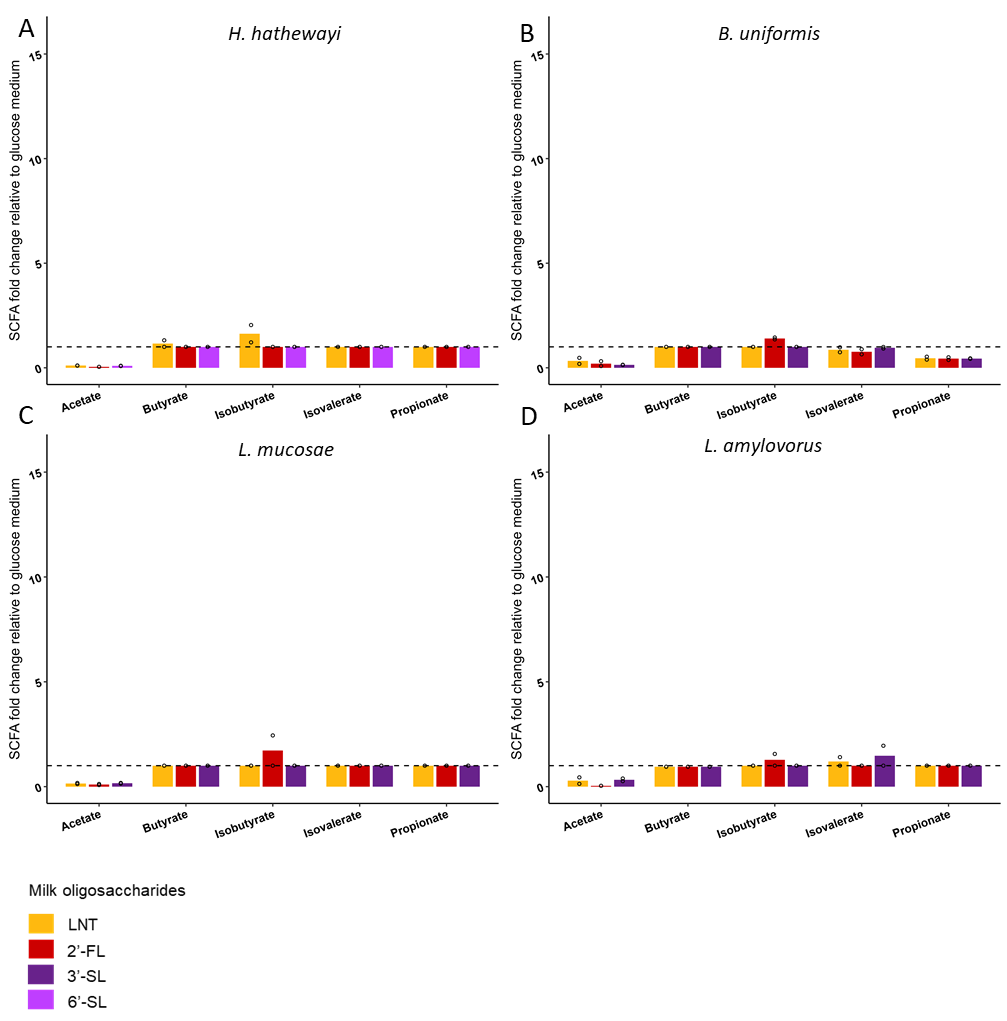


Supplementary Figure 2: In vitro SCFA production by (A) H. hathewayi (B) B. uniformis (C) L. mucosae (D) L. amylovorus cultivated in semi-defined media (mYCFA or mMRS) supplemented with 0.5% lacto-N-Tetraose (LNT), 2'-fucosyllactose (2’-FL), 3’-sialyllactose (3’-SL) or 6'-sialyllactose (6’-SL). The concentrations were measured during the stationary phase. Values were calculated by subtracting the values from semi-defined media without bacterial strains. Data are expressed relative to the concentrations measured in the positive control, histograms represent mean values and dots represent values of each replicate (n = 2 or 3).


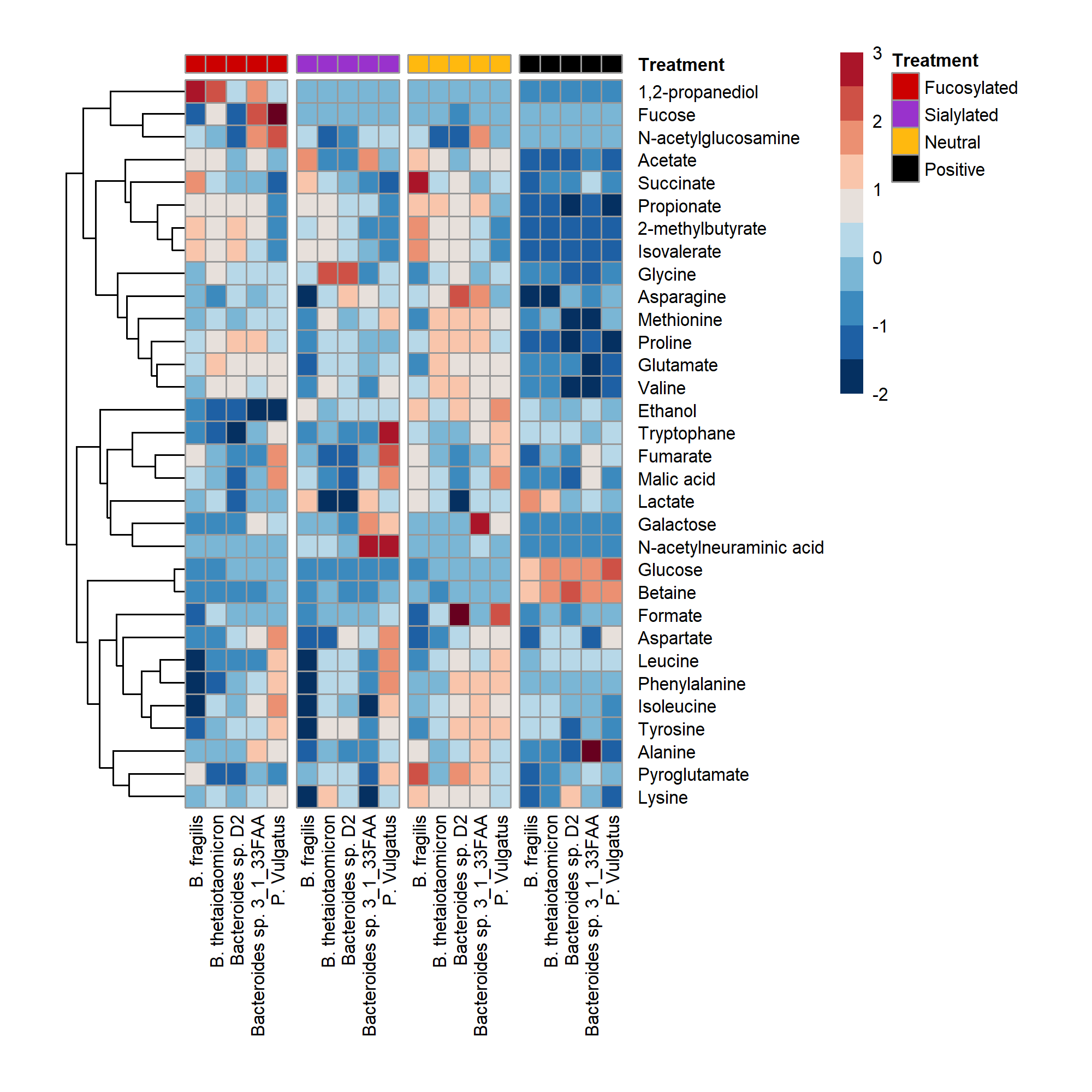


Supplementary Figure 3: Metabolome profiles in bacterial supernatants from isolated bacteria cultivated with glucose or neutral lacto-N-Tetraose (LNT), fucosylated 2'-fucosyllactose (2’-FL) or sialylated 3’-sialyllactose (3’-SL)/6'-sialyllactose (6’-SL) as carbon source. Heatmap representing the mean relative concentration of metabolites (rows) from isolated bacteria cultivated with glucose or MOs as carbon source (columns) in bacterial supernatant. The colors represent the Z-scores (row-scaled relative expression) from low (blue) to high values (red).


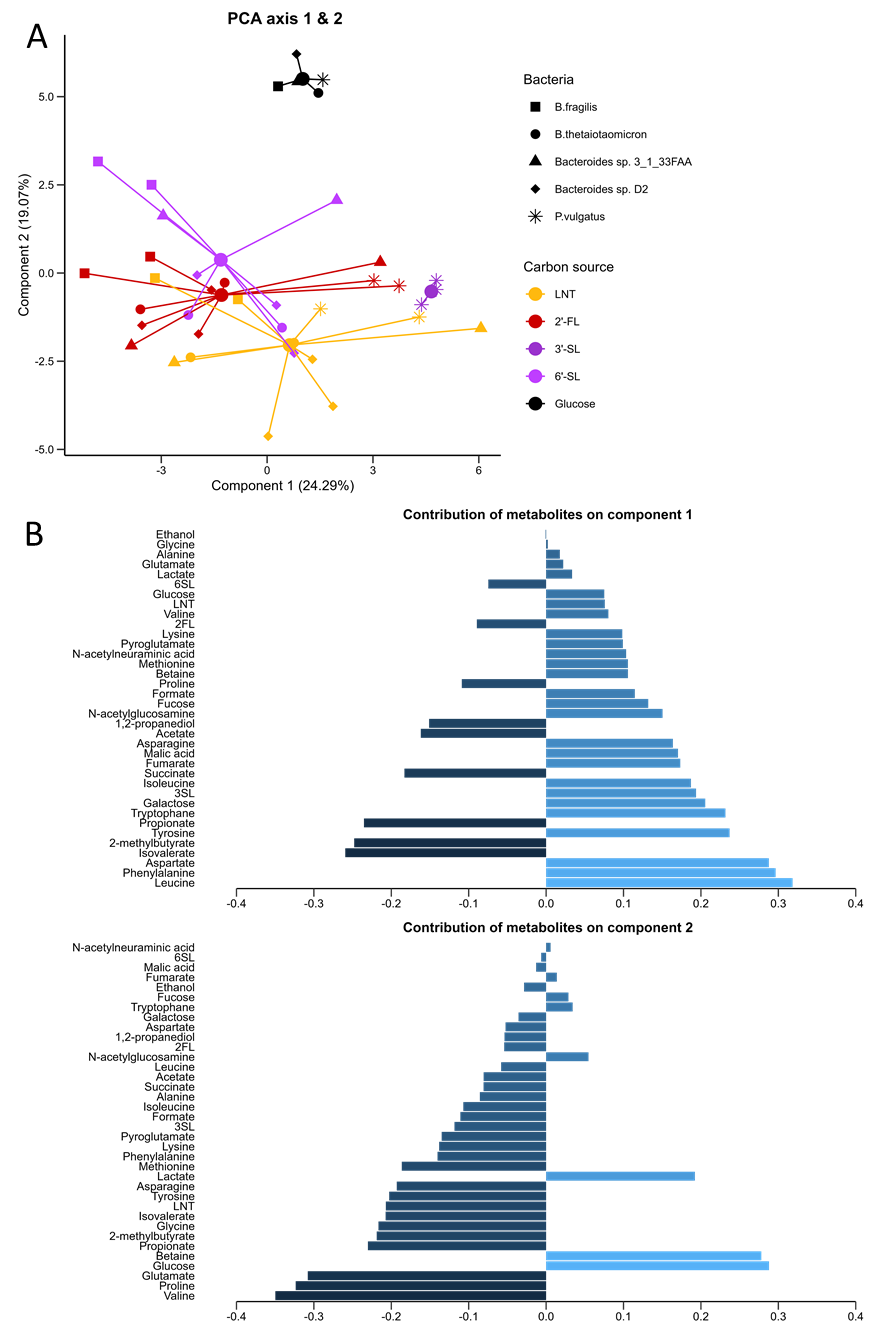


Supplementary Figure 4: Nuclear magnetic resonance-based metabolomics during the stationary phase in supernatants of Bacteroides fragilis, Bacteroides thetaiotaomicron, Bacteroides sp. D2, Bacteroides sp. 3_1_33FAA and Phocaeicola vulgatus cultivated in semi-defined media (mYCFA) supplemented with 0.5% glucose (positive control), LNT, 2’-FL, 3’-SL or 6’-SL. (A) Principal component analysis (PCA) plot built with the relative abundance of each metabolite. (B) Loadings of the first and second component of the PCA. Each coordinate represents a single replicate of one bacterial species cultivated with a sole carbon source (MO or glucose) (n = 2 or 3).
